# Depth-Resolved Three-Dimensional Sound Source Reconstruction Using Acoustic Arrays: Imaging Performance, Localization Error, and Validation

**DOI:** 10.3390/s26195994

**Published:** 2026-09-22

**Authors:** Qi Huang, Wen-Cheng Tang, Jun-Jie Xie, Ru-Xin Lu

**Affiliations:** 1 School of Mechanical Engineering, Southeast University, Nanjing 211189, China; 2 Air Force Logistics Academy, Xuzhou 221000, China

**Keywords:** acoustic beamforming, sound source localization, cross-spectral, delay-and-sum, steering vector, signal processing

## Abstract

Beamforming signal processing based on acoustic microphone arrays is widely used for sound source localization and imaging. However, when investigating sound sources in complex environments, such as cockpit environments or on complex aircraft surfaces, planar imaging becomes less suitable. In addition, the array aperture and microphone density significantly influence imaging capability in different directions, not only in the frontal direction. Therefore, this paper extends the conventional beamforming algorithm into three dimensions, focusing on sound source localization and imaging quality along the depth direction of the array aperture. Theoretically, a cross-spectral matrix and an error function are introduced into the delay-and-sum algorithm to comparatively analyze the imaging quality. The influence of arranging microphones along the depth direction on the system’s imaging performance is investigated, and an experimental validation scheme comparing a planar array with a depth-wise asymmetric array is designed. The beamforming imaging performance in the X-, Y-, and Z-directions is analyzed, including comparisons of main-lobe width, the influence of frequency, and steering-vector-induced error phenomena. Test results indicate that microphone placement along the depth affects imaging performance in the Z-direction, and that appropriate amplitude compensation in the algorithm can effectively control localization errors.

## 1. Introduction

Beamforming results are typically presented as two-dimensional maps of the relative contribution of acoustic power. This two-dimensional imaging mode is highly advantageous for evaluating the performance of new algorithms and usually provides readers with an intuitive representation of performance. However, when the test environment involves complex surfaces—such as vehicle body envelopes [1] or aircraft fuselage envelopes—with large curvature and substantial depth complexity [2], localization and imaging become considerably more difficult. In recent years, many researchers have conducted related studies addressing this issue. Shikha et al. [3] identified the locations of harmonic sound sources in the full three-dimensional space using a limited number of acoustic signals, aiming to address the localization error in 3D space. Chiariotti et al. [4] proposed the average beamforming concept to mitigate ghost sources in spatial acoustic problems. Du et al. [5] proposed a design approach for coding metasurfaces with arbitrarily curved configurations to achieve beamforming in arbitrary directions, thereby providing a three-dimensional solution for beamforming applications. Legg et al. [6] reported that three-dimensional imaging methods suffer from substantially lower spatial resolution in the range direction away from the array than in the lateral directions. Padois et al. [7,8] investigated the influence of perpendicular and parallel scanning planes on imaging performance and compared the performance of three-dimensional and two-dimensional imaging algorithms in terms of source localization, source level estimation, and computational cost. Nevertheless, due to the limitations imposed by the array aperture, the resolution in the depth direction is generally much poorer than the lateral resolution. More importantly, while this depth-resolution degradation has been widely observed, existing studies have treated it primarily as a resolution issue; a systematic and quantitative investigation of the localization bias in the depth direction—in particular, how the choice of steering-vector formulation systematically shifts the estimated source depth—is still lacking. Since at least four steering-vector formulations coexist in the literature and are often used interchangeably, the absence of such an analysis leaves practitioners without guidance on formulation selection and error compensation in depth-resolved 3D imaging. Accordingly, the present study addresses this problem under controlled conditions—a fully anechoic environment with monopole-like sources—so that the steering-vector-formulation and array-geometry effects on depth localization can be isolated from environmental factors such as reflections and source complexity. A quantitative understanding of these effects under such baseline conditions is a prerequisite for the reliable deployment of three-dimensional beamforming in the complex-surface scenarios mentioned above, which constitute the long-term application background rather than the direct subject of this paper.

Among beamforming methods, delay-and-sum (DAS) [9,10] is one of the most classical and fundamental techniques in array signal processing. Similar to other beamforming approaches, the implementation of the DAS relies on the relative geometric relationship between the sound source and the sensors. Regardless of whether the sensors are located in the far field or near field relative to the source, the DAS virtually steers or focuses the array toward a specific direction (plane-wave propagation) or a particular point in space (spherical-wave propagation). By calculating the relative signal delays among sensors and summing them, the algorithm enhances the actual signal source while suppressing unwanted noise. After obtaining the virtual signal data for all reconstructed points, if only a single signal source exists within the reconstruction region, the delayed signals can be considered in phase, thereby maximizing the beamforming output. When focusing on any reconstructed point that does not correspond to the true source location, the signals combine out of phase, resulting in amplitude values lower than those at the true source position. However, this leads to a well-known limitation of the conventional DAS method: the beamforming map is often affected by a relatively large main lobe and significant sidelobes, with sidelobes being a major source of contamination and leakage. Strong sound sources can generate power outputs at locations without actual sources through convolution with sidelobes in the beamforming map, thereby producing the phenomenon of false sources. Consequently, it is often difficult to accurately identify the true source location and strength using beamforming maps obtained by the DAS algorithm. Another notable drawback of the DAS algorithm is that the beamwidth increases as the frequency decreases. To overcome these shortcomings, many researchers have proposed advanced algorithms based on improvements to the DAS approach. Zhai et al. [11] proposed an irregular-grid beamforming method to minimize main-lobe distortion in the beamformer output. Guo et al. [12] proposed an improved masking beamforming method, which can separate acoustic components with spatial characteristics different from those of the target reactor. Xu et al. [13] proposed a continuous acoustic source localization method for composite structures based on a dispersion wave-velocity-modified beamforming approach, which improves localization accuracy. Yang et al. [14] proposed an off-grid deconvolution beamforming method based on the output of DAS, which is less affected by grid spacing, noise interference, and source coherence.

Beamforming based on the cross-spectral matrix (CSM) [15,16,17] is one of the most commonly used acoustic imaging methods. Its output can be regarded as the convolution of the true source strength distribution with a spatial impulse response [18], resulting in significantly improved spatial resolution. Compared with the conventional DAS algorithm, the CSM-based method generally exhibits a narrower main lobe and better sidelobe suppression. The synthetic CSM completion problem can be addressed by exploiting the low-rank property of the matrix and the continuity of the acoustic field [19]. However, even when the acoustic sources are ideal stationary monopoles, interpolation errors are inevitably introduced during the completion of the synthetic CSM. These errors can be readily observed as localization deviations in the reconstructed acoustic images. To further investigate this issue, the multi-dimensional matrix data of the three-dimensional spatial sound pressure reconstructed by DAS-S are incorporated into the CSM framework for processing, so as to analyze the spatial imaging capability of the improved algorithm.

In summary, rather than merely extending DAS and CSM beamforming to three-dimensional space—which by itself follows established practice—this paper focuses on the systematic characterization and control of depth-direction localization errors in 3D acoustic imaging. The main contributions are threefold: (1) The depth localization errors induced by four commonly used steering-vector formulations are theoretically derived and quantitatively compared, revealing that the depth error is a systematic, formulation-dependent bias rather than a mere resolution limitation. (2) A dedicated experimental protocol comparing a planar array with a depth-wise asymmetric array is designed, allowing the influence of microphone placement along the depth direction to be isolated and experimentally verified. (3) A practical error-control strategy is proposed, in which appropriate amplitude compensation suppresses the depth localization bias, and a two-stage workflow (DAS-S pre-localization followed by CSM-EF refinement) balances imaging quality against computational cost.The remainder of this paper is organized as follows. Section 2 introduces the DAS-S, CSM-S, and CSM-EF techniques. Section 3 presents a theoretical analysis of localization errors and four types of steering vectors. Section 4.1 describes the experimental and simulation conditions as well as the two array configurations. Section 4.2 presents three-dimensional imaging cases using different arrays and imaging techniques. Section 4.3 and Section 4.4 provide numerical comparisons of the main-lobe performance. Section 4.5 and Section 4.6 analyze and discuss error phenomena observed in experiments and the influence of steering vectors on system imaging, including both single-source and multi-source tests.

## 2. Method: A Three-Dimensional Delay-and-Sum Beamforming Approach Incorporating a Cross-Spectral Matrix and an Error Function

### 2.1. Three-Dimensional Beamforming Based on DAS

This beamforming approach is developed based on the conventional planar DAS method, extended into three-dimensional space. By performing a layer scan of the focal planes along the Z-axis to identify the X-Y plane that yields the relative maximum output, the depth information of the acoustic source can be extracted, thereby generating a three-dimensional beamforming map. This computational procedure is hereafter referred to as the DAS-S algorithm. As shown in Figure 1, the coordinate origin *O* is defined to establish a Cartesian coordinate system. The virtual plane spacing is defined as ΔZ. A meshed virtual plane is defined as a reconstruction plane, a mesh node is defined as a reconstruction point, and the spacing intervals of adjacent points along the *X*- and *Y*-axes of an equivalent reconstruction plane are defined as ΔX and ΔY, respectively. Acoustic field reconstruction is conducted by calculating the normalized beamforming power output at all reconstruction points, which is also referred to as the acoustic field response. Any reconstruction point in space is defined as $\left(x_{u},y_{v},z_{w}\right)$, where u=1,2,…,U, v=1,2,…,V, and w=1,2,…,W. Here, *u*, *v*, and *w* denote the indices of grid points in the three directions of the reconstruction space, respectively. We assume a microphone array consisting of a total of *B* microphones with coordinates $\left(x_{b},y_{b},z_{b}\right)$, b=1,2,…,B. Then, we designate the microphone denoted by b=1 as the reference microphone with coordinates $\left(x_{0},y_{0},z_{0}\right)$. The pressure signal received at the reference microphone can be defined as a function of the angular frequency ω of the source as follows [20]:

(1) $$P_{0}\left(\omega \right)=\frac{A}{r_{a,0}}e^{-jkr_{a,0}}$$

Here, *A* is the strength of the monopole source, defined as the sound pressure value on the spherical surface 1 m from the center of the sound source. $r_{a0}$ is the distance between the source and the reference microphone, and k=ω/c is the wave number, where *c* is the propagation velocity of sound. The pressure signal of the *b*th microphone will undergo attenuation relative to $P_{0}\left(\omega \right)$, which can be expressed as follows:

(2) $$P_{b}\left(\omega \right)=P_{0}\left(\omega \right)\cdot \frac{r_{a,0}}{r_{a,b}}\cdot e^{-jk\left(r_{a,b}-r_{a,0}\right)}=\frac{A}{r_{a,b}}\cdot e^{-jkr_{a,b}}$$

Here, $r_{a,b}$ is the distance between the $X_{a}$ microphone and the source $X_{b}$. According to the principle of DAS, the complex normalized beamforming pressure output $V_{c}\left(x_{u},y_{v},z_{w},\omega \right)$ relative to the actual output at the reconstruction point *X* is given as follows:

(3) $$\begin{array}{c} V_{c}\left(x_{u},y_{v},z_{w},\omega \right)=\frac{1}{B}\sum_{b=1}^{B}\left[\xi_{b}P_{b}\left(\omega \right)\cdot e^{-jk\left(r_{c,0}-r_{c,b}\right)}+P_{e}\left(\omega \right)\right]\\ =\frac{1}{B}\sum_{b=1}^{B}\left(\xi_{b}\frac{A}{r_{a,b}}\cdot e^{-j\omega \left[\frac{r_{a,b}}{c}+\frac{\left(r_{c,0}-r_{c,b}\right)}{c}\right]}+P_{e}\left(\omega \right)\right) \end{array}$$

Above, $\xi_{b}$ denotes the weighting coefficient of microphone $X_{b}$ in the DAS operation, and $P_{e}\left(\omega \right)$ represents the ambient white noise, which can be modeled as a zero-mean random variable. Since $r_{a,b}$ is independent of the output value at the reconstruction point, the above equation reveals that the phase component determines the maximum output of the DAS-S method. Specifically, $V_{c}\left(x_{u},y_{v},z_{w},\omega \right)$ achieves a relative maximum output when the position of the reconstruction point coincides with that of the acoustic source. To facilitate subsequent matrix computations, the steering vector $u_{b}^{I}$ for the aforementioned DAS method is defined as follows:

(4) $$u_{b}^{I}=\frac{1}{B}e^{-jk\left(r_{c,b}-r_{c,0}\right)}$$

The introduction of the steering vector enables a clearer formulation of the DAS process. The basic idea of DAS is to adjust the phase of the sound signal $P_{b}\left(\omega \right)$ received by each microphone through the steering vector $u_{b}^{I}$, such that the signals are coherently focused onto a new reconstruction point. The resulting output $V_{c}\left(x_{u},y_{v},z_{w},\omega \right)$ can be organized into a data matrix $A^{I}\in C^{U\times V\times W}$, from which the acoustic source contribution map is obtained. The source contribution map generated by conventional beamforming can highlight the dominant sound-pressure contribution while suppressing interfering noise to a certain extent. In addition, the weighting in the DAS process is designed according to the relative propagation distance between the source and microphones. In the frequency domain, this is equivalent to applying an appropriate phase-shift factor. Therefore, the conventional beamforming result essentially reflects the relative distribution of sound-pressure contributions, rather than the exact physical solution of the source field. The DAS-S method effectively addresses this limitation by performing layer-by-layer scanning along the source-depth direction, thereby producing *W* matrix slices, each of size $C^{U\times V}$. By comparing the mean contribution of each slice, the depth location of the source can be identified. Hence, the fundamental strategy of DAS-S is localization first, followed by imaging. It should be noted that the steering vector $u_{b}^{I}$ compensates only the phase term, without amplitude correction. Both theoretical analysis and experimental results indicate that this setting is reasonable for ensuring depth localization accuracy. However, it also introduces new issues. Therefore, a new steering vector $u_{b}^{\mathrm{II}}$ is introduced in the subsequent CSM-S analysis.

### 2.2. Cross-Spectral Matrix-Enhanced DAS-S

The output of the cross-spectral method can be regarded as the convolution of the actual source intensity distribution and the spatial impulse response [19,21]. Consequently, by integrating the cross-spectral solution with the result $V_{c}\left(x_{u},y_{v},z_{w},\omega \right)$ derived from DAS-S, a novel fusion algorithm, designated as CSM-S, is developed to represent the average power spectral output $I_{c}\left(x_{u},y_{v},z_{w},\omega \right)$ under three-dimensional slicing conditions, as expressed below:

(5) $$I_{c}\left(x_{u},y_{v},z_{w},\omega \right)=\frac{1}{B^{2}}\sum_{m,n=1}^{B}R_{nm}e^{-j\omega \left[\tau_{m}\left(x_{u},y_{v},z_{w}\right)-\tau_{n}\left(x_{u},y_{v},z_{w}\right)\right]}$$

where ${\left(\cdot \right)}^{*}$ denotes the complex conjugate, $t\left(x_{u},y_{v},z_{w}\right)=\frac{r_{c,0}-r_{c,b}}{c}$ represents the time delay at the reconstruction point, and $R_{nm}\;=\;P_{m}\left(\omega \right)P_{n}^{*}\left(\omega \right)$ denotes the cross-spectral element. For convenience, in subsequent computation and representation, the above problem and the DAS-S method are expressed in matrix form. First, for Equation (2), the acoustic pressure signals $P_{b}\left(\omega \right)$ received by all microphones can be represented by a column vector **h**, as shown below:

(6) $$h=A\cdot \left[\begin{array}{cccc} \xi_{1} & 0 & \cdots & 0\\ 0 & \xi_{2} & 0 & 0\\ \vdots & 0 & \ddots & 0\\ 0 & 0 & 0 & \xi_{b} \end{array}\right]\left[\begin{array}{c} \frac{1}{r_{1,a}}e^{-jkr_{1,a}}\\ \frac{1}{r_{2,a}}e^{-jkr_{2,a}}\\ \vdots \\ \frac{1}{r_{b,a}}e^{-jkr_{b,a}} \end{array}\right].$$

In the later simulations, the delay terms and weighting coefficient matrix ξ can be assumed to be the identity matrix E. Subsequently, the cross-spectral matrix can be expressed as

(7) $$R=hh^{H},$$

where ${\left(\cdot \right)}^{H}$ denotes the Hermitian transpose of a matrix. The vector $h^{H}\in C^{1\times B}$ and the cross-spectral matrix $R\in C^{B\times B}$ are given as follows:

(8) $$h^{H}=A\cdot \left[\begin{array}{cccc} \frac{1}{r_{1,a}}e^{jkr_{1,a}} & \frac{1}{r_{2,a}}e^{jkr_{2,a}} & \ldots & \frac{1}{r_{b,a}}e^{jkr_{b,a}} \end{array}\right],$$

(9) $$R=A^{2}\cdot \left[\begin{array}{cccc} \frac{1}{r_{1,a}\cdot r_{1,a}} & \frac{1}{r_{1,a}\cdot r_{2,a}}e^{-jk\left(r_{1,a}-r_{2,a}\right)} & \ldots & \frac{1}{r_{1,a}\cdot r_{b,a}}e^{-jk\left(r_{1,a}-r_{b,a}\right)}\\ \frac{1}{r_{2,a}\cdot r_{1,a}}e^{-jk\left(r_{2,a}-r_{1,a}\right)} & \frac{1}{r_{2,a}\cdot r_{2,a}} & \ldots & \frac{1}{r_{2,a}\cdot r_{b,a}}e^{-jk\left(r_{2,a}-r_{b,a}\right)}\\ \vdots & \vdots & \ddots & \vdots \\ \frac{1}{r_{b,a}\cdot r_{1,a}}e^{-jk\left(r_{b,a}-r_{1,a}\right)} & \frac{1}{r_{b,a}\cdot r_{2,a}}e^{-jk\left(r_{b,a}-r_{2,a}\right)} & \ldots & \frac{1}{r_{b,a}\cdot r_{b,a}} \end{array}\right].$$

The steering vector $u_{\mathrm{II}}$ can also be expressed as

(10) $$u_{\mathrm{II}}=\frac{1}{B}\left[\frac{r_{1,c}}{r_{0,c}}e^{-jk\left(r_{1,c}-r_{0,c}\right)},\frac{r_{2,c}}{r_{0,c}}e^{-jk\left(r_{2,c}-r_{0,c}\right)},\ldots ,\frac{r_{b,c}}{r_{0,c}}e^{-jk\left(r_{b,c}-r_{0,c}\right)}\right].$$

Substituting the above h and $u^{H}$ into Equations (3) and (5), the matrix-form expressions of $V_{c}\left(x_{u},y_{v},z_{w},\omega \right)$ and the average power spectral output $I_{c}\left(x_{u},y_{v},z_{w},\omega \right)$ can be obtained as

(11) $$\left\{\begin{array}{c} V_{c}\left(x_{u},y_{v},z_{w},\omega \right)=\left|u^{H}h\right|\\ I_{c}\left(x_{u},y_{v},z_{w},\omega \right)=u^{H}\mathrm{Ru}=u^{H}hh^{H}u \end{array}\right..$$

Equations (7)–(9) describe the ideal rank-one CSM of a single stationary monopole and are used for the analytical derivation and the numerical simulations. In the experiments, the CSM at each test frequency is estimated from the calibrated pressure time histories $x_{b}\left[\cdot \right]$ of the *B* microphones as

(12) $$\hat{R}\left(\omega \right)=p\left(\omega \right)\;p^{H}\left(\omega \right),\;p_{b}\left(\omega \right)=\frac{1}{S_{w}}\sum_{n=0}^{N_{\mathrm{FFT}}-1}w\left[n\right]\;x_{b}\left[n_{0}+n\right]\;e^{-j\omega n/f_{s}},$$

where w[n] is a Hanning window of length $N_{\mathrm{FFT}}$ with $S_{w}=\sum_{n}w\left[n\right]$, $f_{s}$ is the sampling frequency, and $n_{0}$ is a common starting sample for all channels, so that the inter-channel phase relations are preserved. Equation (12) is a single-snapshot estimate (K=1 block); the full matrix including the diagonal is retained, and it is Hermitian positive-semidefinite. This choice is adequate for the deterministic, stationary pure-tone excitation and the anechoic environment used here (Section 4.1). In all experimental results, $\hat{R}$ replaces R in Equations (11) and (17)–(20); the processing parameters are listed in Section 4.3.

### 2.3. Cross-Spectral Matrix–Enhanced Difference Function

The cross-spectral example based on the difference function method can likewise be extended to the three-dimensional depth direction, hereafter referred to as CSM-EF [22,23].

This algorithm is constructed on the basis of the cross-spectral matrix **R**. First, a simulated cross-spectral element is assumed as $R_{nm}^{\prime }=P_{m}^{\prime }\left(\omega \right)\;P_{n}^{\prime *}\left(\omega \right)$. It can be observed that the structure of $R_{nm}^{\prime }$ is similar to that of $R_{nm}$ in Equations (2)–(6). The form of $P_{m}^{\prime }\left(\omega \right)$ is the same as that in Equations (2) and (3), except that the specific reference is replaced by the reconstruction point in the $X_{c}$ direction. The explicit expression is given as follows:

(13) $$P_{b}^{\prime }\left(\omega \right)=\frac{A}{r_{c,b}}\cdot e^{-jkr_{c,b}},$$

where $P_{b}^{\prime }\left(\omega \right)$ corresponds to $P_{m}^{\prime }\left(\omega \right)$ and $P_{n}^{\prime }\left(\omega \right)$, where the definitions of *m* and *n* are the same as those given in Equation (5). Therefore, the simulated cross-spectral unit can be expressed a

(14) $${R_{nm}}^{\prime }={P_{m}}^{\prime }\left(\omega \right){P_{n}}^{\prime *}\left(\omega \right)=A^{2}\cdot \frac{e^{-jk\left({r_{m}}^{\prime }-{r_{n}}^{\prime }\right)}}{{r_{m}}^{\prime }\cdot {r_{n}}^{\prime }},$$

Here, $r^{\prime }=r_{c,b}$. In matrix form, $R_{nm}^{\prime }$ corresponds to $R^{\prime }$, and $P_{b}^{\prime }\left(\omega \right)$ corresponds to $h_{c}$:

(15) $$h_{c}=A\cdot \left[\begin{array}{c} \frac{1}{r_{1,c}}e^{-jkr_{1,c}}\\ \frac{1}{r_{2,c}}e^{-jkr_{2,c}}\\ \vdots \\ \frac{1}{r_{b,c}}e^{-jkr_{b,c}} \end{array}\right],$$

(16) $$R^{\prime }=A^{2}\cdot \left[\begin{array}{cccc} \frac{1}{r_{1,c}\cdot r_{1,c}} & \frac{1}{r_{1,c}\cdot r_{2,c}}e^{-jk\left(r_{1,c}-r_{2,c}\right)} & \ldots & \frac{1}{r_{1,c}\cdot r_{b,c}}e^{-jk\left(r_{1,c}-r_{b,c}\right)}\\ \frac{1}{r_{2,c}\cdot r_{1,c}}e^{-jk\left(r_{2,c}-r_{1,c}\right)} & \frac{1}{r_{2,c}\cdot r_{2,c}} & \ldots & \frac{1}{r_{2,c}\cdot r_{b,c}}e^{-jk\left(r_{2,c}-r_{b,c}\right)}\\ \vdots & \vdots & \ddots & \vdots \\ \frac{1}{r_{b,c}\cdot r_{1,c}}e^{-jk\left(r_{b,c}-r_{1,c}\right)} & \frac{1}{r_{b,c}\cdot r_{2,c}}e^{-jk\left(r_{b,c}-r_{2,c}\right)} & \ldots & \frac{1}{r_{b,c}\cdot r_{b,c}} \end{array}\right],$$

After comparing the the measured CSM R with the simulated CSM $R^{\prime }=aH$, where H contains only geometric quantities, $H_{nm}=e^{-jk(r_{m}^{\prime }-r_{n}^{\prime })}/\left(r_{m}^{\prime }r_{n}^{\prime }\right)$, and $a=A^{2}$ is a real, nonnegative scale parameter representing the squared source strength, the CSM-EF objective at the reconstruction point is defined as the scalar least-squares erro:

(17) $$E_{c}\left(x_{u},y_{v},z_{w},\omega \right)=\sum_{m,n=1}^{B}{\left|R_{nm}-aH_{nm}\right|}^{2}={∥R-aH∥}_{F}^{2},$$

where ${∥\cdot ∥}_{F}$ denotes the Frobenius norm. Introducing the Frobenius inner product ${\langle X,Y\rangle }_{F}=\mathrm{tr}\left(X^{H}Y\right)=\sum_{m,n}X_{nm}^{*}Y_{nm}$, Equation (17) expands as

(18) Ec(a)=∥R∥F2−2aRe〈H,R〉F+a2∥H∥F2.

Writing $H=gg^{H}$ with $g_{b}=e^{-jkr_{c,b}}/r_{c,b}$, and noting that the measured CSM R is Hermitian positive-semidefinite, one has ${\langle H,R\rangle }_{F}=\mathrm{tr}\left(gg^{H}R\right)=g^{H}Rg\geq 0$, i.e., the inner product is real and nonnegative. Hence, Equation (18) is a real-coefficient quadratic in the single real variable *a*, its unconstrained minimizer automatically satisfies a≥0, and setting $\partial E_{c}/\partial a=0$ gives the closed-form optimum

(19) $$a^{*}=\frac{{\langle H,R\rangle }_{F}}{{∥H∥}_{F}^{2}},\;E_{c}^{\min}={∥R∥}_{F}^{2}-\frac{{\langle H,R\rangle }_{F}^{2}}{{∥H∥}_{F}^{2}}.$$

Since ${∥R∥}_{F}^{2}$ depends only on the measured data and not on the reconstruction point, minimizing $E_{c}^{\min}$ over the scan grid is equivalent to maximizing $F={∥R∥}_{F}^{2}-E_{c}^{\min}$, which defines the output of the CSM-EF method:

(20) $$F\left(x_{u},y_{v},z_{w},\omega \right)=\frac{{\langle H,R\rangle }_{F}^{2}}{{∥H∥}_{F}^{2}}=\frac{{\left[\mathrm{tr}(H^{H}R)\right]}^{2}}{\mathrm{tr}(H^{H}H)}=\frac{{\left|\sum_{m,n=1}^{B}R_{nm}\;\frac{e^{\;jk\left(r_{m}^{\prime }-r_{n}^{\prime }\right)}}{r_{m}^{\prime }\;r_{n}^{\prime }}\right|}^{2}}{\sum_{m,n=1}^{B}\frac{1}{{\left(r_{m}^{\prime }\;r_{n}^{\prime }\right)}^{2}}}.$$

The equivalence between Equation (20) and the direct numerical minimization of Equation (17) is verified in Appendix A.

## 3. Discussion of Localization Errors in the Depth Direction of the 3D Localization Plane Based on Spherical Waves

### 3.1. Localization Error Sources and Identification Criteria

In the experiments and numerical simulations, it was observed that localization errors may occur under certain conditions, particularly in the depth direction [24]. This phenomenon is mainly caused by the mismatch introduced by different steering-vector formulations. For example, the steering vector $u_{I}$ compensates only for the phase term, whereas the steering vector $u_{\mathrm{II}}$ compensates for both the phase and amplitude terms. In this section, four commonly used steering vectors are introduced, and their influence on localization accuracy is analyzed. Since the focus of this study is source localization, a monopole source located at $X_{a}\;\left(x_{a},y_{a},z_{a}\right)$ is considered. The complex acoustic pressure measured at the microphone positions can be written as

(21) $$P\left(x_{b}\right)=a\left(x_{a},x_{b}\right)q\left(x_{a}\right)$$

where $P(x_{b})$ denotes the acoustic pressure at the microphone locations, $q(x_{a})$ denotes the source strength of the monopole source, and $a(x_{a},x_{b})$ represents the steering vector from the source to the microphones. The complex output at the reconstruction point $x_{c}$ is defined as

(22) $$V_{c}\left(x_{c}\right)=u{\left(x_{c}\right)}^{H}p$$

where $V_{c}\left(x_{c}\right)$ denotes the complex-domain output at the reconstruction point, $u(x_{c})$ is the steering vector associated with the reconstruction point, and p is the measured complex pressure vector. Based on the CSM formulation, the beamforming output can be expressed as

(23) $$I_{c}\left(x_{c}\right)=R\left\{V_{c}\left(x_{c}\right)V_{c}{\left(x_{c}\right)}^{*}\right\}=u{\left(x_{c}\right)}^{H}pp^{H}u\left(x_{c}\right)=u{\left(x_{c}\right)}^{H}\mathrm{Ru}\left(x_{c}\right),$$

where $R=pp^{H}$ is the cross-spectral matrix. Since the reconstruction point $x_{c}={\left(x_{c},y_{c},z_{c}\right)}^{T}$ is three-dimensional, the requirement that $I_{c}$ attain a maximum at the true source position $x_{a}$ implies the first-order necessary condition

(24) $$\nabla_{x_{c}}I_{c}\left(x_{c}\right){|}_{x_{c}=x_{a}}=0,\;\nabla_{x_{c}}={\left[\frac{\partial }{\partial x_{c}},\frac{\partial }{\partial y_{c}},\frac{\partial }{\partial z_{c}}\right]}^{T}.$$

Equation (24) is necessary but not sufficient. A steering vector is therefore said to be localization-consistent if (i) Equation (24) holds and (ii) $x_{a}$ is in addition a maximizer of $I_{c}$. Conversely, if the gradient at $x_{a}$ is nonzero, then $x_{a}$ is not even a stationary point and hence cannot be a local maximum; the peak of the beamforming map is necessarily displaced from the true source position.

#### Common Framework

For a monopole source, the transfer function from a point x to microphone *b* is

(25) $$a_{b}\left(x\right)=\frac{1}{r_{b}\left(x\right)}\;e^{-jkr_{b}\left(x\right)},\;r_{b}\left(x\right)=∥x-x_{b}∥,$$

and we abbreviate $r_{c,b}=r_{b}\left(x_{c}\right)$, $r_{a,b}=r_{b}\left(x_{a}\right)$, $a_{c}=a\left(x_{c}\right)$, $a_{a}=a\left(x_{a}\right)$. With $p=q\;a_{a}$, the complex output of Equation (22) reads

(26) $$V_{c}\left(x_{c}\right)=q\sum_{b=1}^{B}u_{b}^{*}\left(x_{c}\right)\;a_{b}\left(x_{a}\right),\;I_{c}\left(x_{c}\right)=V_{c}V_{c}^{*}={\left|V_{c}\left(x_{c}\right)\right|}^{2}.$$

Because $I_{c}$ is real, its gradient follows from the product rule as

(27) ∇xcIc=Vc*∇xcVc+Vc∇xcVc*=2ReVc*∇xcVc.

All four steering vectors considered below depend on $x_{c}$ only through the *B* distances $r_{c,1},\ldots ,r_{c,B}$. These distances are not independent coordinates but functions of the single variable $x_{c}$, so the chain rule gives

(28) $$\nabla_{x_{c}}V_{c}=\sum_{m=1}^{B}\frac{\partial V_{c}}{\partial r_{c,m}}\;\nabla_{x_{c}}r_{c,m}=\sum_{m=1}^{B}\frac{\partial V_{c}}{\partial r_{c,m}}\;e_{c,m},\;e_{c,m}=\frac{x_{c}-x_{m}}{r_{c,m}},$$

where $e_{c,m}$ is the unit vector pointing from microphone *m* to the focus point. The partial derivatives with respect to the distances thus enter only as scalar coefficients; the spatial gradient is their vector sum weighted by $e_{c,m}$. At $x_{c}=x_{a}$, one has $r_{c,m}=r_{a,m}$ and $e_{c,m}=e_{a,m}=\left(x_{a}-x_{m}\right)/r_{a,m}$. One geometric fact will be used repeatedly. For a planar array lying in the plane z=0 and a source at $z_{a}>0$ (the configuration used throughout this work), every unit vector $e_{a,m}$ has a strictly positive depth component, $e_{a,m}\cdot \hat{z}=z_{a}/r_{a,m}>0$. Hence, any combination $\sum_{m}w_{m}e_{a,m}$ with strictly positive weights $w_{m}$ has a strictly positive depth component and is therefore nonzero.

### 3.2. Steering Vector I

Steering vector I is the most basic formulation, compensating only the phase component from each microphone to the reconstruction point. It corresponds to $u_{I}$ in the DAS-S, as shown in Equation (4):

(29) $$u_{I,b}=\frac{1}{B}\frac{a_{b}\left(x_{c}\right)}{|a_{b}\left(x_{c}\right)|}=\frac{1}{B}e^{-jkr_{c,b}}.$$

Substituting Equation (29) into Equation (26) yields

(30) $$V_{c}\left(x_{c}\right)=\frac{q}{B}\sum_{b=1}^{B}\frac{1}{r_{a,b}}e^{jk(r_{c,b}-r_{a,b})},\;\frac{\partial V_{c}}{\partial r_{c,m}}=\frac{q}{B}\;\frac{jk}{r_{a,m}}\;e^{jk(r_{c,m}-r_{a,m})}.$$

At $x_{c}=x_{a}$, all phase factors reduce to unity, so that $V_{c}\left(x_{a}\right)=\frac{q}{B}\sum_{b}r_{a,b}^{-1}$ and, by Equation (28), $\nabla_{x_{c}}V_{c}\left(x_{a}\right)=\frac{jkq}{B}\sum_{m}r_{a,m}^{-1}e_{a,m}$. The product $V_{c}^{*}\nabla V_{c}$ is purely imaginary, and Equation (27) gives

(31) ∇xcIc|xc=xa=2|q|2B2∑b1ra,bRejk∑mea,mra,m=0,

so the criterion in Equation (24) is satisfied. That $x_{a}$ is moreover a maximum follows globally from the triangle inequality applied to Equation (30):

(32) $$I_{c}\left(x_{c}\right)=\frac{{\left|q\right|}^{2}}{B^{2}}|\sum_{b=1}^{B}\frac{1}{r_{a,b}}e^{jk(r_{c,b}-r_{a,b})}{|}^{2}\leq \frac{{\left|q\right|}^{2}}{B^{2}}{\left(\sum_{b=1}^{B}\frac{1}{r_{a,b}}\right)}^{2}=I_{c}\left(x_{a}\right),$$

with equality if and only if all phases $k(r_{c,b}-r_{a,b})$ coincide modulo 2π, which holds at $x_{c}=x_{a}$. The true source position is therefore a global maximizer of $I_{c}$, and the vanishing gradient in Equation (31) is the corresponding first-order condition.

### 3.3. Steering Vector II

Steering vector II compensates not only the phase delay but also the amplitude component. It was introduced by Mosher [25] and Brooks [26]. The CSM-S method described in Section 2.2 employs this steering vector

(33) $$u_{\mathrm{II},b}=\frac{1}{B}\frac{a_{b}\left(x_{c}\right)}{|a_{b}\left(x_{c}\right){|}^{2}}=\frac{1}{B}r_{c,b}\;e^{-jkr_{c,b}}.$$

Note that, compared with Equation (10), the common complex factor $e^{jkr_{0,c}}/r_{0,c}$ associated with the reference distance $r_{0,c}$ is omitted here without loss of generality: it is identical for all array elements at a given focus point and merely rescales the output. Substituting Equation (33) into Equation (26),

(34) $$V_{c}\left(x_{c}\right)=\frac{q}{B}\sum_{b=1}^{B}\frac{r_{c,b}}{r_{a,b}}e^{jk(r_{c,b}-r_{a,b})},\;\frac{\partial V_{c}}{\partial r_{c,m}}=\frac{q}{B}\;\frac{1+jk\;r_{c,m}}{r_{a,m}}\;e^{jk(r_{c,m}-r_{a,m})}.$$

at $x_{c}=x_{a}$, $V_{c}\left(x_{a}\right)=q$ and $\partial V_{c}/\partial r_{c,m}=\frac{q}{B}\;\left(r_{a,m}^{-1}+jk\right)$. Inserting these into Equations (27) and (28), the imaginary part jk drops out upon taking the real part and one obtains

(35) $$\nabla_{x_{c}}I_{c}{|}_{x_{c}=x_{a}}=\frac{{2|q|}^{2}}{B}\sum_{m=1}^{B}\frac{e_{a,m}}{r_{a,m}}=\frac{{2|q|}^{2}}{B}\sum_{m=1}^{B}\frac{x_{a}-x_{m}}{r_{a,m}^{2}}.$$

This is a sum of unit vectors pointing from each microphone towards the source, with strictly positive weights $1/(B\;r_{a,m})$. For the one-sided planar array considered here, its depth component, $\partial I_{c}/\partial z_{c}{|}_{x_{a}}=\frac{{2|q|}^{2}}{B}\sum_{m}z_{a}/r_{a,m}^{2}>0$, is strictly positive. Hence, $\nabla I_{c}\left(x_{a}\right)\neq 0$, the criterion in Equation (24) is violated, and $x_{a}$ is not a stationary point of $I_{c}$, let alone a maximum. Moreover, since the gradient points away from the array, the output increases as the focus point moves beyond the true source depth, so the peak of the beamforming map is displaced along the depth direction. The transverse components of Equation (35), $\sum_{m}\left(x_{a}-x_{m}\right)/r_{a,m}^{2}$ and $\sum_{m}\left(y_{a}-y_{m}\right)/r_{a,m}^{2}$, contain contributions from both signs and largely cancel for a source near the axis of a centro-symmetric array, which is why the displacement is observed predominantly in depth. The physical origin of this displacement can be understood by inspecting the two competing factors that constitute each term of Equation (32): an amplitude weight $r_{c,b}/r_{a,b}$ introduced by the amplitude compensation, and a phase-coherence term $e^{jk(r_{c,b}-r_{a,b})}$. The phase-coherence factor is maximized at the actual source position, where all microphone contributions add in phase; however, when the focus point moves along the depth direction, the path-length differences ${\dot{x}}_{b}-{\dot{y}}_{b}$ among the microphones vary only slowly, so the resulting loss of coherence is of second order (a consequence of the intrinsically weak axial resolution of spherical-wave beamforming). In contrast, the amplitude weight $r_{c,b}/r_{a,b}$ grows monotonically and to first order with the focus distance, with slope $1/{\dot{y}}_{b}$ at the actual source position-precisely the weight $1/r_{a,m}$ appearing in Equation (35). Consequently, at $x_{c}=x_{a}$, the coherence loss is locally stationary while the amplitude weighting still provides a positive gradient, so the total output continues to increase as the focus point moves away from the array. The maximum response therefore settles at a depth where the first-order amplitude gain is balanced by the accumulated coherence loss, i.e., at a position displaced from the actual source along the depth direction. In the transverse directions, the phase mismatch grows much faster, which explains why the displacement is observed mainly in depth. It should be emphasized that this displacement does not contradict the correct power quantification obtained at the actual source position. Since the amplitude compensation exactly inverts the propagation attenuation, $u_{\mathrm{II}}^{H}a\left(x_{a},x_{b}\right)=1$ holds at $x_{c}=x_{a}$, and hence $I_{c}\left(x_{a}\right)=R\left\{q\left(x_{a}\right)q{\left(x_{a}\right)}^{*}\right\}$, in agreement with the reference source power. The estimated power at the true position and the location of the maximum response are governed by different properties of the beamforming map: the former depends on the value of $I_{c}$ at $x_{c}=x_{a}$, whereas the latter depends on where the gradient of $I_{c}$ vanishes. Amplitude compensation preserves the former but destroys the latter.

### 3.4. Steering Vector III

The third steering vector, $u_{\mathrm{III}}$ [27], is designed to recover the source power without loss, i.e.,

(36) Ic(xc=xa)=Re{q(xa)q(xa)*}.

Under this constraint, the response to spatially white noise is minimized. It is given by

(37) $$u_{\mathrm{III}}=\frac{a_{c}}{a_{c}^{H}a_{c}},\;u_{\mathrm{III},b}=\frac{e^{-jkr_{c,b}}}{r_{c,b}\;S\left(x_{c}\right)},\;S\left(x_{c}\right)=a_{c}^{H}a_{c}=\sum_{i=1}^{B}\frac{1}{r_{c,i}^{2}}.$$

Substituting into Equation (26) gives

(38) $$V_{c}\left(x_{c}\right)=q\;\frac{N(x_{c})}{S(x_{c})},\;N\left(x_{c}\right)=a_{c}^{H}a_{a}=\sum_{b=1}^{B}\frac{e^{jk(r_{c,b}-r_{a,b})}}{r_{c,b}\;r_{a,b}},$$

with

(39) $$\frac{\partial N}{\partial r_{c,m}}=\frac{1}{r_{a,m}}\left(-\frac{1}{r_{c,m}^{2}}+\frac{jk}{r_{c,m}}\right)e^{jk(r_{c,m}-r_{a,m})},\;\frac{\partial S}{\partial r_{c,m}}=-\frac{2}{r_{c,m}^{3}}.$$

At $x_{c}=x_{a}$, $N=S=S_{a}\equiv \sum_{i}r_{a,i}^{-2}$, so $V_{c}\left(x_{a}\right)=q$ (consistent with Equation (36)) and, by the quotient rule,

(40) $$\frac{\partial V_{c}}{\partial r_{c,m}}{|}_{x_{a}}=\frac{q}{S_{a}}\left(\frac{\partial N}{\partial r_{c,m}}-\frac{\partial S}{\partial r_{c,m}}\right)=\frac{q}{S_{a}}\left(\frac{1}{r_{a,m}^{3}}+\frac{jk}{r_{a,m}^{2}}\right).$$

Equations (27) and (28) then yield

(41) $$\nabla_{x_{c}}I_{c}{|}_{x_{c}=x_{a}}=\frac{{2|q|}^{2}}{S_{a}}\sum_{m=1}^{B}\frac{e_{a,m}}{r_{a,m}^{3}}=\frac{{2|q|}^{2}}{\sum_{i}r_{a,i}^{-2}}\sum_{m=1}^{B}\frac{x_{a}-x_{m}}{r_{a,m}^{4}}.$$

As for steering vector II, this is a positively weighted sum of the unit vectors $e_{a,m}$; its depth component $\frac{{2|q|}^{2}}{S_{a}}\sum_{m}z_{a}/r_{a,m}^{4}$ is strictly positive for a one-sided planar array. The criterion in Equation (24) is therefore violated and the maximum of the map is displaced away from the array, by the same first-order mechanism as for steering vector II (the weights decay as $r_{a,m}^{-3}$ instead of $r_{a,m}^{-1}$, but their sign is unchanged).

### 3.5. Steering Vector IV

Steering vector IV has been applied by Wang [28] and Suzuki [29,30]. It is the unit-norm normalization of $a_{c}$, $u_{\mathrm{IV}}^{H}u_{\mathrm{IV}}=1/B$:

(42) $$u_{\mathrm{IV}}=\frac{1}{\sqrt{B}}\frac{a_{c}}{\sqrt{a_{c}^{H}a_{c}}},\;u_{\mathrm{IV},b}=\frac{e^{-jkr_{c,b}}}{r_{c,b}\sqrt{B\;S(x_{c})}},$$

so that, with *N* and *S* as in Equations (37) and (38),

(43) $$V_{c}\left(x_{c}\right)=\frac{q}{\sqrt{B}}\frac{N(x_{c})}{\sqrt{S(x_{c})}},\;I_{c}\left(x_{c}\right)=\frac{{\left|q\right|}^{2}}{B}\frac{|a_{c}^{H}a_{a}{|}^{2}}{a_{c}^{H}a_{c}}.$$

The maximum property follows directly from the Cauchy-Schwarz inequality,

(44) $$I_{c}\left(x_{c}\right)\leq \frac{{\left|q\right|}^{2}}{B}\;a_{a}^{H}a_{a}=I_{c}\left(x_{a}\right),$$

with equality if and only if $a_{c}\propto a_{a}$, which holds at $x_{c}=x_{a}$. Hence, $x_{a}$ is a global maximizer. For consistency with the gradient criterion, we also evaluate Equation (28) directly: at $x_{a}$,

(45) $$\frac{\partial V_{c}}{\partial r_{c,m}}{|}_{x_{a}}=\frac{q}{\sqrt{BS_{a}}}\left(\frac{\partial N}{\partial r_{c,m}}-\frac{1}{2}\frac{\partial S}{\partial r_{c,m}}\right)=\frac{q}{\sqrt{BS_{a}}}\;\frac{jk}{r_{a,m}^{2}},$$

which is purely imaginary relative to $V_{c}\left(x_{a}\right)=q\sqrt{S_{a}/B}$, so that ∇xcIc(xa)=2Re{Vc*∇Vc}=0, in agreement with Equation (44). Comparing Equation (45) with Equation (40) shows precisely where the two normalizations differ: the factor $\frac{1}{2}\partial S/\partial r_{c,m}$ of the square-root normalization exactly cancels the real part $-1/r_{a,m}^{3}$ arising from $\partial N/\partial r_{c,m}$, whereas the full factor $\partial S/\partial r_{c,m}$ of steering vector III over-compensates it and leaves the residual $+1/r_{a,m}^{3}$ in Equation (41).

#### Summary

The four results can be written in the common form

(46) $$\nabla_{x_{c}}I_{c}{|}_{x_{c}=x_{a}}=2{\left|q\right|}^{2}\sum_{m=1}^{B}w_{m}\;e_{a,m},\;w_{m}^{I}=w_{m}^{\mathrm{IV}}=0,\;w_{m}^{\mathrm{II}}=\frac{1}{B\;r_{a,m}},\;w_{m}^{\mathrm{III}}=\frac{r_{a,m}^{-3}}{\sum_{i}r_{a,i}^{-2}}.$$

For steering vectors I and IV, the gradient vanishes and, by Equations (32) and (44), $x_{a}$ is a global maximizer. For steering vectors II and III, the weights are strictly positive, so, for a one-sided planar array, the gradient has a strictly positive depth component; $x_{a}$ is not a stationary point and the peak is displaced away from the array. The existence of the bias is thus a first-order effect that does not require a Hessian; the curvature of $I_{c}$ only determines the magnitude of the displacement, which is quantified numerically in Section 4.6.

## 4. Simulation Results and Discussion

### 4.1. Experimental and Simulation Test Setup

The sound source experiment was conducted in a fully anechoic chamber of dimensions 3m×3m×3m, with a lower cutoff frequency of 63Hz. The background noise level inside the chamber is below −18dB(A) (re 20μPa), i.e., lower than the threshold of human hearing, as certified in the chamber qualification measurements. In the experiment, a small loudspeaker was used to approximate a monopole sound source. The loudspeaker was driven by stepped sinusoidal tones from 1kHz to 6kHz in steps of 500Hz. Each tone was gated by a rectangular on/off envelope with a repetition period of 10s. For each test case, a record of 5.0625s (165,888 samples per channel) was acquired within the steady-state portion of the tone on a 60-channel PULSE front-end, from which the 27 channels corresponding to the microphone positions in Table 1 and Table 2 were extracted. The CSM was estimated from a single $N_{\mathrm{FFT}}$-sample block taken from the central part of the record according to Equation (12) (Table 3); block averaging was not required because the excitation is a deterministic stationary tone recorded in an anechoic environment. The complete experiment both arrays, all source positions and all 11 test frequencies was carried out three times; the results reported below are from one of these runs. The amplitude and phase information of the acoustic signals was acquired by two different arrays, as shown in Figure 2. In Figure 2a, the Brüel & Kjær WA-1558 wheel array was used, while Figure 2b shows the experimental configuration employing a self-developed array with microphones distributed along the depth direction. These two arrays are hereafter referred to as array-I and array-II, respectively. The microphone positions are illustrated in Figure 3a,b.

The experiment was designed to verify the reliability of the numerical simulations by comparing the performance of a two-dimensional array with that of a three-dimensional array. The two-dimensional array was based on the 60-channel wheel array manufactured by Brüel & Kjær (Nærum, Denmark; model WA-1558).

The 27 microphone positions were arranged in accordance with the standard topology-optimized placement scheme provided in the official Brüel & Kjær documentation for the WA-1558 sliced wheel array [31], which preserves the azimuthal coverage, the spoke structure, and the full 0.48 m aperture of the original 60-channel layout. The exact coordinates of all microphones are listed in Table 1, and the same configuration was adopted in the numerical simulations. The center of the array was defined as the origin of the reference Cartesian coordinate system. For the three-dimensional array configuration, microphones of the same type (Type 4958) were used to enable a fair comparison with the wheel array. Its diameter was also 0.48 m, consistent with that of the wheel array.

For the reconstruction configuration, the observation plane was defined as parallel to the *X*-*Y* plane. By scanning planes along the depth direction, a complete reconstruction volume covering both the front and rear regions of the array was obtained. The reconstruction plane extended from −0.5m to 0.5m in both the *X* and *Y* directions, with a grid spacing of 0.01m, resulting in 10,201 scanning points on each plane. The *Z* axis was defined as the depth direction, with its positive direction pointing toward the opening of array-II. The scanning range along the *Z* direction was from −4m to 4m, with a grid spacing of 0.25m. The assumed monopole source was placed within the aforementioned reference coordinate system. The frequency range was 1–6kHz, with a frequency interval of 500Hz. The reference sound pressure level was uniformly set to 50dB.

### 4.2. Comparison of Slice Imaging Results Obtained with Different Algorithms and Arrays

This section presents the three-dimensional imaging performance of the two arrays using three algorithms (DAS-S, CSM-S, and CSM-EF). The experimental results obtained under the same configuration are shown on the right for reference. Due to unavoidable placement tolerances in the practical experiments, the actual source positions deviate slightly from the nominal position. The actual source coordinates, physically measured with respect to the array center prior to data acquisition, are (0.02,−0.1,2) for the array-I setup and (−0.01,−0.06,2) for the array-II setup; these measured values, rather than the beamforming outputs, serve as the ground truth for evaluating localization accuracy. In the simulations, the analysis of localization accuracy and resolution indicates that all three imaging methods successfully reconstruct and identify the source location, in good agreement with the predefined source position. A comparison of the results for array-I and array-II shows that, when the source depth exceeds 1.5 m, the main-lobe width of array-II is slightly larger than that of array-I. A comparison of Figure 4(a-1,b-1) indicates that array-I provides better sidelobe suppression than array-II. Comparing Figure 4(c-1,c-2) reveals that, in the experimental CSM-S results obtained with array-I, the global maximum of the output map does not coincide with the true source position. It should be emphasized that no assumption about the source location is made at any stage during the processing, and all algorithms are applied to the same raw dataset with an identical processing chain. This spurious peak is a deterministic consequence of the sidelobe structure of the focused beam pattern in array-I in the considered frequency band, rather than the result of any assumption on the source position. As shown in Equation (11), the CSM-S output satisfies $I_{c}\left(x_{u},y_{v},z_{w},\omega \right)=u^{H}Ru={\left|u^{H}h\right|}^{2}$, so spurious maxima occur wherever the steering vector of a scan point is strongly correlated with the measured pressure vector h generated by the true source, i.e., at the secondary maxima (sidelobes) of the array point-spread function. For the planar array-I, all microphones lie in the array plane ($z_{b}=0$ for b=1,…,B, where $\left(x_{b},y_{b},z_{b}\right)$ denotes the position of microphone $X_{b}$). Consider scan points located on the depth axis passing through the source $X_{a}$. Under the Fresnel approximation, $r_{c,b}\approx z_{w}+\rho_{b}^{2}/\left(2z_{w}\right)$, where $\rho_{b}=\sqrt{{\left(x_{b}-x_{a}\right)}^{2}+{\left(y_{b}-y_{a}\right)}^{2}}$ is the in-plane distance between microphone $X_{b}$ and the projection of the source onto the array plane. After the removal of the delay common to all channels, the differential propagation path of microphone $X_{b}$ reduces to the wavefront-curvature term $\Delta_{b}\left(z_{w}\right)=\rho_{b}^{2}\;\left(1/z_{w}-1/z_{a}\right)/2$, so that the depth sensitivity of the beamformer rests solely on this weak quadratic term, and the magnitude of the coherent summation $S\left(z_{w}\right)=|\sum_{b=1}^{B}e^{jk\Delta_{b}\left(z_{w}\right)}|$ exhibits a secondary maximum at the depth at which the phase increments $k\Delta_{b}\left(z_{w}\right)$ over the quasi-regular radial distribution $\left\{\rho_{b}^{2}\right\}$ of array-I approach multiples of 2π and the channels re-add nearly in phase. Since this depth scales with the wavenumber *k*, the secondary lobe enters the scanning volume only within 3500–4500 Hz, which explains the frequency selectivity of the artifact; the amplitude weighting of the steering vector $u_{\mathrm{II}}$ employed by CSM-S further modifies the relative level of this secondary lobe along the depth direction, consistent with the extremum analysis of steering vector II in Section 3, which shows that the amplitude compensation distorts the depth response of the beamforming map. In the idealized simulations, this secondary lobe remains below the true main lobe; in the experiments, reflections and channel mismatch raise the sidelobe floor so that it exceeds the true peak in the CSM-S map, whereas the difference-function optimization of CSM-EF (Equation (20)), applied to exactly the same raw dataset, penalizes sidelobe contributions and suppresses the artifact. For array-II, the nonzero microphone offsets $z_{b}$ add a term linear in $z_{w}$ to $\Delta_{b}\left(z_{w}\right)$, which destroys the coherent summation at the secondary maximum. A comparison of the imaging results of the three algorithms shows that CSM-EF provides the most accurate source contribution map, featuring the narrowest main lobe and the best sidelobe suppression. In addition, Figure 4(b-1,d-1,f-1), as well as the corresponding experimental results, each exhibit a single distinct main lobe, whereas the corresponding results obtained with array-I exhibit two lobes. The interfering lobe appears symmetrically with respect to the true main lobe along the depth direction, forming a mirror image. This observation indicates that the asymmetric microphone distribution of array-II along the *z*-axis can effectively suppress pseudo-sources in practical applications and eliminate the associated interference.

Figure 5 presents the depth-slice maps around the source location. The *X*-*Z* plane passing through the source location is selected to compare the imaging performance of the two arrays at different depths. The operating frequency is f=1500Hz. The parameter for each column is indicated at the top of the figure, corresponding to source depths of $z_{0}=0.5\;m$, 1m, 1.5m, and 2m, respectively. The algorithm used in each row, together with the corresponding array, is labeled on the left-hand side. Under the same algorithm, array-I exhibits better imaging performance than array-II at source depths of 0.5m and 1m. However, as the source depth increases, the single-source imaging results of the two arrays become increasingly similar. This trend is more evident when the CSM-EF method is employed, and is further discussed in the subsequent quantitative analysis of the main-lobe width. In addition, Figure 5 compares the imaging results obtained using the DAS-S and CSM-EF algorithms. The results show that the CSM-EF algorithm, which incorporates the cross-spectral matrix and difference-function optimization, achieves higher imaging resolution, as evidenced by reduced sidelobe interference and a narrower main-lobe width. To quantitatively characterize the trade-off between imaging accuracy and computational efficiency, Table 4 summarizes the computation times of DAS-S, CSM-S, and CSM-EF under identical reconstruction conditions, i.e., the same single-source configuration, the same frequency, the same scanning region, and the same hardware and software platform (with the CPU Intel core i5-6500 and 8 Gbytes of RAM). Two three-dimensional scanning grids containing 85,833 and 418,241 grid points are considered, and the reported values are the averages of 〈3〉 independent runs.

As shown in Table 4, DAS-S is two to three orders of magnitude faster than the two CSM-based methods, because it only involves delay-and-sum operations on the time-domain (or frequency-domain) signals of the individual channels. In contrast, the computational costs of CSM-S and CSM-EF are comparable and are both dominated by the construction of the cross-spectral matrix and the point-by-point steering-vector evaluation over the three-dimensional grid; the additional difference-function optimization of CSM-EF introduces only a marginal overhead. For all three methods, the computation time increases approximately linearly with the number of grid points (the grid is refined by a factor of about 4.9, and the computation times of the CSM-based methods increase by a factor of about 4.4–5.0). The choice of array geometry (array-I versus array-II) has a negligible influence on the computational cost, since the two arrays contain the same number of microphones. Therefore, CSM-EF achieves the best imaging performance among the investigated methods at a computational cost essentially identical to that of CSM-S, whereas DAS-S remains preferable for applications in which real-time processing outweighs the demand for high spatial resolution. It should be noted that, although the aperture, sensor count, weighting, and processing were matched between the two arrays and their X-Y projections were made as similar as possible, the projected microphone positions are not strictly identical. The present comparison should therefore be regarded as a preliminary qualitative verification of the benefit of depth-wise microphone placement; a rigorously controlled experiment in which only the Z offsets are varied on a dedicated rig is planned as future work. The strong dependence of the depth-direction imaging performance on the source depth admits a simple physical interpretation in terms of the array aperture *D*, the acoustic wavelength λ, the Z-direction microphone extent $L_{z}$, and the source array distance $z_{0}$. In focused beamforming, depth discrimination relies on the wavefront curvature sampled across the array, whose maximum path-length difference over an aperture *D* is approximately $D^{2}/\left(8z_{0}\right)$. Consequently, while the lateral resolution scales as $\Delta x∼\lambda z_{0}/D$, the depth resolution degrades quadratically with distance, $\Delta z∼\kappa \;\lambda \;{\left(z_{0}/D\right)}^{2}$, and effective depth focusing is restricted to the near field $z_{0}≲D^{2}/\lambda$. For the present aperture D=0.48 m, this limiting distance is approximately 1.0 m at 1.5 kHz and 3.0 m at 4.5 kHz, which is consistent with the rapid growth of the Z-direction HPBW with source depth observed in Figure 6 and Figure 7, with the convergence of the two arrays for $z_{0}\geq 1.5$ m at 1.5 kHz in Figure 5, and with the low-frequency depth-localization deviations analyzed in Section 4.5. The Z-direction microphone distribution in array-II ($L_{z}\approx 0.21$ m, cf. Table 2) contributes an additional direct depth baseline whose relative benefit scales as $L_{z}/z_{0}$: at $z_{0}=0.5$ m ($L_{z}/z_{0}\approx 0.4$), this yields the observed 36% reduction in the Z-direction HPBW, whereas at $z_{0}=2$ m ($L_{z}/z_{0}\approx 0.1$) the benefit vanishes and the slightly less efficient X-Y projection of array-II results in the observed 8% increase of the main-lobe width. It should therefore be emphasized which aspects of the observed behavior are general and which are configuration-specific. The following trends are intrinsic to three-dimensional focused beamforming and independent of the particular algorithm: (i) the quadratic degradation of depth resolution with $z_{0}/D$ and its proportionality to λ; (ii) the existence of a crossover depth-governed by $L_{z}$ and by the near-field limit $D^{2}/\lambda$-beyond which depth-distributed microphones no longer improve the depth resolution; and (iii) the suppression of the mirror pseudo-source by array-II, which is a purely geometric consequence of breaking the front-back symmetry of a planar array and holds for any nonzero Z-direction offset. By contrast, the specific quantitative values reported here (the 36% HPBW reduction at $z_{0}=0.5$ m, the crossover near $z_{0}\approx 1.5$ m, and the 8% penalty at $z_{0}=2$ m) are tied to the present aperture, Z-direction extent, microphone count, and frequency range, and would scale accordingly for other array geometries.The relationship between the main-lobe width and the depth-localization error, as well as the conditions under which systematic localization bias arises, are discussed in Section 4.5.

### 4.3. Comparison of the Main-Lobe Widths of CSM-S and DAS-S for the Two Arrays

To evaluate the performance of the localization techniques in determining source positions, an additional parameter, the half-power beam width (HPBW) [32,33], is introduced to quantify the main-lobe width. The HPBW is defined as the width of the main lobe at which the beamforming output drops by 3 dB (half power) below its peak value. For each spatial direction, the HPBW is measured along a one-dimensional profile extracted from the three-dimensional beamforming map through the peak position, parallel to the *X*-, *Y*-, or *Z*-axis. Since the half-power level generally falls between two grid points, the two −3 dB crossing positions on each profile are determined by linear interpolation between the adjacent grid points bracketing the half-power level, and the HPBW is taken as the distance between the two crossings. In this paper, the relative HPBW is defined as $n=\mathrm{HPB}W_{\mathrm{CSM}\;\text{-}\;S}/\mathrm{HPB}W_{1}$, where ${\mathrm{HPBW}}_{\mathrm{CSM}\text{-}S}$ is the main-lobe width of CSM-S and ${\mathrm{HPBW}}_{1}$ is the main-lobe width of DAS-S obtained with array-I. The main-lobe width of DAS-S with array-I is taken as the reference to improve the readability of the data and to facilitate the comparison of the different algorithms. The relative HPBW *n* of CSM-S is shown in Figure 6. The scatter points denote the experimental results (one measurement per point) and the solid curves the simulation results. The shaded areas are the 95% confidence bands of trend curves fitted to the experimental points; they indicate the uncertainty in the fitted trend only and do not represent the variability of repeated measurements. In Figure 6c,f, points at which the DAS-S reference width exceeds the scanning region (${\mathrm{HPBW}}_{1}>2.6$ m) are absent because *n* is undefined; in Figure 6f, the point $z_{0}=2.0$ m, 4000 Hz is absent because the CSM-S width exceeds the scanning region (n>117%). Absolute values of all *Z*-direction widths, including the censored cases, are given in Table A4, Table A5 and Table A6. A single source is assumed at A=(X,Y)=(0.04,−0.08)m for both arrays and both algorithms (DAS-S and CSM-S), with source depths $z_{0}=$0.5 m, 1.0 m, 1.5 m, and 2.0 m. The frequency ranges from 1 to 6kHz with an interval of 500 Hz. Figure 6a–c show the relative HPBW obtained with array-I in the *X*-, *Y*-, and *Z*-directions. In the *X*- and *Y*-directions, the fluctuation of *n* is small, approximately 3%, and is largely unaffected by changes in frequency and depth. The frequency can be characterized by the Helmholtz number $\mathrm{He}=f\cdot \frac{d}{c}$ (where *d* and *c* are the array aperture and the speed of sound, respectively), and the high-frequency limit is set to He=4 [34]. Since the aperture of both arrays is 0.48 m, the corresponding frequency is 2720 Hz. As shown in Figure 6c, each curve exhibits a downward trend at low frequencies, whereas, in the *X*- and *Y*-directions, the curves are smooth and nearly linear with small amplitudes, as shown in Figure 6a,b. This implies that, compared with DAS-S, CSM-S achieves better main-lobe control in the *Z*-direction than in the *X*- and *Y*-directions at low frequencies. Additionally, in Figure 6c, at a few low-frequency points (f≤〈1500〉 Hz for $z_{0}=\langle 1.5\rangle$ m and 〈2.0〉 m), the main lobe of CSM-S in the Z-direction was so wide that the −3 dB crossing could not be determined within the scanning region, i.e., only a lower bound HPBW>1 m could be established. Since no valid HPBW value can be assigned to these right-censored points, they are omitted from Figure 6c (normally, each curve contains 12 measurement points). It should be noted that, under the same conditions, the corresponding HPBW of DAS-S is even larger, so this omission does not bias the conclusion that n<100%; if anything, it makes the reported low-frequency comparison more conservative. Figure 6d-f show the relative HPBW obtained with array-II in the *X*-, *Y*-, and *Z*-directions. Because the denominator of *n* is identical in all subfigures, the left and right columns can be directly compared to evaluate the relative HPBW of the two arrays. As the source depth decreases, the relative HPBW of array-II in the *X*- and *Y*-directions also decreases. This effect is more pronounced in the *Z*-direction: when $z_{0}=0.5$ m, *n* is reduced by approximately 35%. However, it is worth noting that, when $z_{0}=2$ m, the value of *n* for array-II is 8% larger than that for array-I. For array-II, the curves in the *Y*-direction are slightly higher (by approximately 2-9%) than those in the *X*-direction.

In summary, for array-I, all resolved data points lie below 100%, and, at every censored point at which CSM-S is resolved, the bound n<HPBW/2.6 m is likewise below 100%; CSM-S therefore yields a smaller main-lobe width than DAS-S wherever the comparison can be made. In particular, in the low-frequency range, CSM-S achieves better main-lobe control in the *Z*-direction than DAS-S. Regarding the comparison between the two arrays, when the source depth is less than 1.5 m, array-II effectively reduces the main-lobe width, whereas for $z_{0}=2.0$ m it is inferior to array-I and, at 4000 Hz, even to the DAS-S reference (n>117%, Table A5). Compared with the *X*- and *Y*-directions, the HPBW of array-II in the *Z*-direction is more sensitive to depth variation: when $z_{0}=0.5$ m, the main-lobe width decreases by 36%.

### 4.4. Comparison of the Main-Lobe Widths of CSM-EF and DAS-S for the Two Arrays

This section compares CSM-EF with DAS-S and further quantifies the main-lobe performance of CSM-EF for the two arrays in the *X*-, *Y*-, and *Z*-directions. The same measurement method as in the previous section is applied here. The same treatment of the confidence bands and of the censored reference applies; Figure 7c,f contain the same 11, 10, 8 and 5 points as Figure 6c. The absolute *Z*-direction widths of CSM-EF are listed in Table A6: at seven of the ten censored reference points, CSM-EF with array-I is resolved (upper bounds n<49–86%), and, unlike CSM-S, CSM-EF with array-II is resolved at all points at which the reference is resolved, so that no n>100% case occurs. The vertical axis range is uniformly set to 20–70%. In this section, the parameter *n* is redefined as $n=\mathrm{HPB}W_{\mathrm{CSM}\text{-}\mathrm{EF}}/\mathrm{HPB}W_{1}$, where ${\mathrm{HPBW}}_{\mathrm{CSM}\text{-}\mathrm{EF}}$ is the main-lobe width of CSM-EF and ${\mathrm{HPBW}}_{1}$ is the main-lobe width of DAS-S obtained with array-I. For array-I, shown in Figure 7a–c, the curves follow essentially the same trend as those in Figure 6, but with smaller values of *n*: the relative HPBW is reduced by approximately 20% compared with the CSM-S results. For array-II, shown in Figure 7d–f, *n* is smaller when the source is closer to the array, particularly in the *Z*-direction; however, the main lobe becomes larger when the source depth exceeds 2 m. All other comparisons between the two arrays are essentially similar to those in the previous section. However, it is worth noting that all values of *n* in this section are below 65%, whereas those in Figure 6 exceed this value. This indicates that CSM-EF provides better main-lobe control than either CSM-S or DAS-S.

### 4.5. Frequency-Dependent Localization Error: Tests and Results

Inaccurate localization is commonly observed in experimental tests. The theoretical analysis of the four steering vectors presented in Section 3 provides a reasonable explanation for this issue. In the CSM-S algorithm, steering vector $u_{\mathrm{II}}$ is adopted, which introduces an amplitude compensation term into the steering-vector formulation. This compensation causes a shift between the actual source position and the point of maximum output, which in turn leads to localization errors. Figure 8 presents the single-source imaging results of the CSM-S algorithm based on steering vector $u_{\mathrm{II}}$ (denoted as CSM-u_II_). The results from 2 to 12kHz are analyzed: the first column shows the three-dimensional imaging results, the second column presents the *X*-*Y* localization plane, and the third column shows the *Y*-*Z* localization plane. For all frequency tests, the source is located at (0.04,−0.08,2)m.

As shown in Figure 8, as the frequency increases, the estimated source position gradually approaches the predetermined source location. This trend is particularly evident in the depth direction. A comparison of the *Y*-*Z* localization images in the third column shows that the region of maximum output moves downward as the frequency increases. By comparing the coordinates of the measured maximum-output point with the predetermined source coordinates, the relative error is obtained, as shown in Figure 9.

In Figure 9, the red dashed line represents the maximum allowable error within the current reconstruction space, σ=0.8m. Any value exceeding this limit is regarded as invalid. The maximum errors in the *X*-and *Y*-directions are both within 0.03 m and approach zero at frequencies above 6kHz. In contrast, the error in the *Z*-direction exceeds this limit when the frequency is below 5kHz. As the frequency increases, the error gradually decreases, and the curve becomes increasingly stable at higher frequencies. Two remarks are in order regarding the errors reported in Figure 9. First, the systematic bias analyzed above originates exclusively from the amplitude-compensation term of steering-vector formulation II (and likewise III, cf. Section 4.6); it is not a property of the CSM-based processing itself. For the criterion-satisfying formulations (I and IV) employed in all results in Section 4.2, Section 4.3 and Section 4.4, no such bias exists, and the peak position coincides with the true source position within the grid resolution (0.01m) in the *X*- and *Y*-directions at all tested depths and frequencies. Second, a residual peak deviation nevertheless remains in the *Z*-direction at low frequencies and large source depths, where the main lobe is broad and flat-topped (cf. the *Z*-direction HPBW in Figure 6 and Figure 7): the discrete output maximum may then fall anywhere within the half-power region of the lobe, so that this deviation is bounded by approximately half the *Z*-direction HPBW, reaching up to 0.8m in the worst case observed here and decreasing rapidly with increasing frequency. This deviation is a direct manifestation of the limited depth resolution rather than an algorithmic bias. Consequently, the depth-localization capability of the two arrays and the three methods is quantified in this work through the *Z*-direction HPBW: the reduction in the *Z*-direction HPBW achieved by array-II and the CSM-based methods (up to 36% at $z_{0}=0.5\;m$, Section 4.3 and Section 4.4) translates into a proportional reduction in the worst-case depth-localization error.

### 4.6. Imaging Tests and Error Characteristics of the Four Steering Vectors

The theoretical analysis in Section 3 established the characteristics of the four commonly used steering vectors, denoted I-IV. In the following tests, these four steering vectors were incorporated into the conventional CSM-S algorithm, yielding four corresponding variants: CSM-u_I_, CSM-u_II_, CSM-u_III_, and CSM-u_IV_. Three-dimensional localization and sound-pressure contribution-map analyses were then performed for a single point source. The test frequency was 6kHz. The simulated sound source was located at (0.04,−0.08,2), with source strength $I_{0}$ set such that $I_{0}^{2}=2500$ (arbitrary units, proportional to the acoustic power of the source). The beamforming output at each scan point $x_{c}$ is the quadratic (power-like) quantity $I_{c}^{2}$, and the map is normalized as

$$B_{c}=10\log_{10}\frac{I_{c}^{2}}{I_{\max}^{2}},$$

where $I_{\max}^{2}$ denotes the maximum output over the map and $B_{c}$ is the normalized output level (in dB relative to the map maximum). The four variants were tested, and the outputs at the assumed source position are listed in Table 5.

Table 5 shows that the results obtained using steering vector II and steering vector III give $I_{c}^{2}=2500$, identical to the reference source strength $I_{0}^{2}$. However, the position $x_{c}=x_{a}$ does not correspond to the location of the maximum output. In contrast, steering vectors I and IV show the opposite behavior: their values of $I_{\max}^{2}$ and $I_{c}^{2}$ are identical, yielding $B_{c}=0$ dB. Figure 10 presents the source imaging results on the *Y*-*Z* localization plane, and Figure 11 shows those on the *X*-*Y* localization plane. The normalized output levels at key points are indicated numerically. In the figures, the symbol “∘” denotes the spatial location of the maximum output, while “×” indicates the projection of the assumed source position onto the localization plane.

As shown in the two groups of imaging results above (on the *X*-*Y* and *Y*-*Z* localization planes), the maximum-output positions obtained with steering vectors $u_{I}$ and $u_{\mathrm{IV}}$ coincide with the predetermined source location, both reaching 0 dB. In contrast, those obtained with steering vectors $u_{\mathrm{II}}$ and $u_{\mathrm{III}}$ deviate from the predetermined source location: the deviation is relatively large on the *Y*-*Z* localization plane, reaching 0.5 m, and is 0.022 m on the *X*-*Y* plane. Hence, the criterion in Equation (24) is not satisfied.

Next, a multi-source experiment was conducted to evaluate the imaging performance for multiple sources with different strengths. The algorithm was again the CSM-S algorithm combined with the four steering vectors. The four sources were located at (0.04,−0.04,2), (−0.04,−0.08,2), (0.04,−0.08,2), and (0.04,0.04,2). The reference source strengths were $I_{0}^{2}=2500$, 1600, 900, and 400 (a.u.), corresponding to relative levels of 0, −1.94, −4.44, and −7.96 dB, and the source frequency was 6000 Hz. The four sources were mutually incoherent: independent random phases were assigned to each source in every snapshot, so that the inter-source cross terms of the cross-spectral matrix vanish in expectation. This configuration can be regarded as four point sources of different strengths distributed on the Z=2 m plane. The imaging results on the *X*-*Y* localization plane are analyzed in detail, as shown in Figure 12. The normalized output levels at key points are labeled numerically in the figure, where the symbol “∘” denotes the spatial position of the maximum output, and “×” represents the projection of the predetermined source location onto the localization plane.

The localization-plane images shown above correspond to the plane on which the maximum output is obtained. Steering vectors $u_{I}$ and $u_{\mathrm{IV}}$ localize the sources on the Z=2 m plane, with no error in the depth direction. The peak positions of the four sources coincide with the predetermined source locations, consistent with the single-source results. In contrast, steering vectors $u_{\mathrm{II}}$ and $u_{\mathrm{III}}$ localize the sources on the Z=2.66 m plane. The peak positions of all sources deviate from the predetermined source locations, and thus the criterion in Equation (24) is again not satisfied.

In summary, the results of both the single-source and multi-source tests are consistent with the theoretical analysis of steering vectors I-IV. Steering vectors I and IV satisfy the criterion, with maxima occurring at the predetermined source locations. For steering vectors II and III, although the outputs at the predetermined source locations equal the reference source strength, the maximum-output condition is not fulfilled, so the spatial maxima of the output deviate from the predetermined source locations, resulting in localization errors.

## 5. Conclusions

A beamforming algorithm for sound source depth measurement was designed, and an experimental scheme for validating sound source localization was established. A planar array and a three-dimensional array optimized in the depth direction were employed for comparison. Imaging performance along the X-, Y-, and Z-directions of the three-dimensional coordinate system was analyzed. The tests included comparative slice imaging of a single- and multi-source extended along the depth direction, comparison of the main-lobe proportion in the depth direction, and analysis of imaging localization capability with varying frequency. Finally, the localization error observed during the experiments was analyzed. The results show that, when the sound source depth is shallow, the imaging capability of array-II (which possesses structural characteristics in the Z direction) is clearer, particularly in the depth direction. In terms of algorithm comparison, CSM-EF provided the best sound source map but required longer computation time, especially when the number of grid points was large. Therefore, in scenarios with a large scanning space, the sound source can first be pre-localized using DAS-S, after which CSM-EF can be applied for three-dimensional imaging within a smaller reconstruction region to improve computational efficiency. Finally, the scope and limitations of the present experimental validation should be stated explicitly. All experiments in this work were conducted in a fully anechoic chamber using loudspeaker-driven, monopole-like tonal sources; this configuration was deliberately chosen so that the steering-vector-formulation and array-geometry effects on depth localization could be isolated from environmental factors. Consequently, the performance of the proposed array-algorithm combinations in reflective (non-anechoic) environments and for more complex sources, such as broadband noise and dipole-type sources, remains to be experimentally verified. Two observations from the present study are nevertheless encouraging in this respect. First, the mirror pseudo-source exhibited by the planar array (Section 4.2) is structurally analogous to the image sources introduced by reflecting boundaries, so the pseudo-source suppression capability of array-II demonstrated here is expected to be advantageous in reverberant conditions. Second, since all CSM-based processing in this work operates on a per-frequency-bin basis, broadband sources can in principle be handled bin by bin without any modification of the formulations, and the frequency-dependent behavior characterized in Section 4.3, Section 4.4 and Section 4.5 indicates the performance that such bin-wise processing would inherit at each frequency. A systematic experimental campaign in a semi-reverberant environment, together with tests on broadband and dipole-type sources, is planned as the immediate next step of this research.

## Figures and Tables

**Figure 1:**
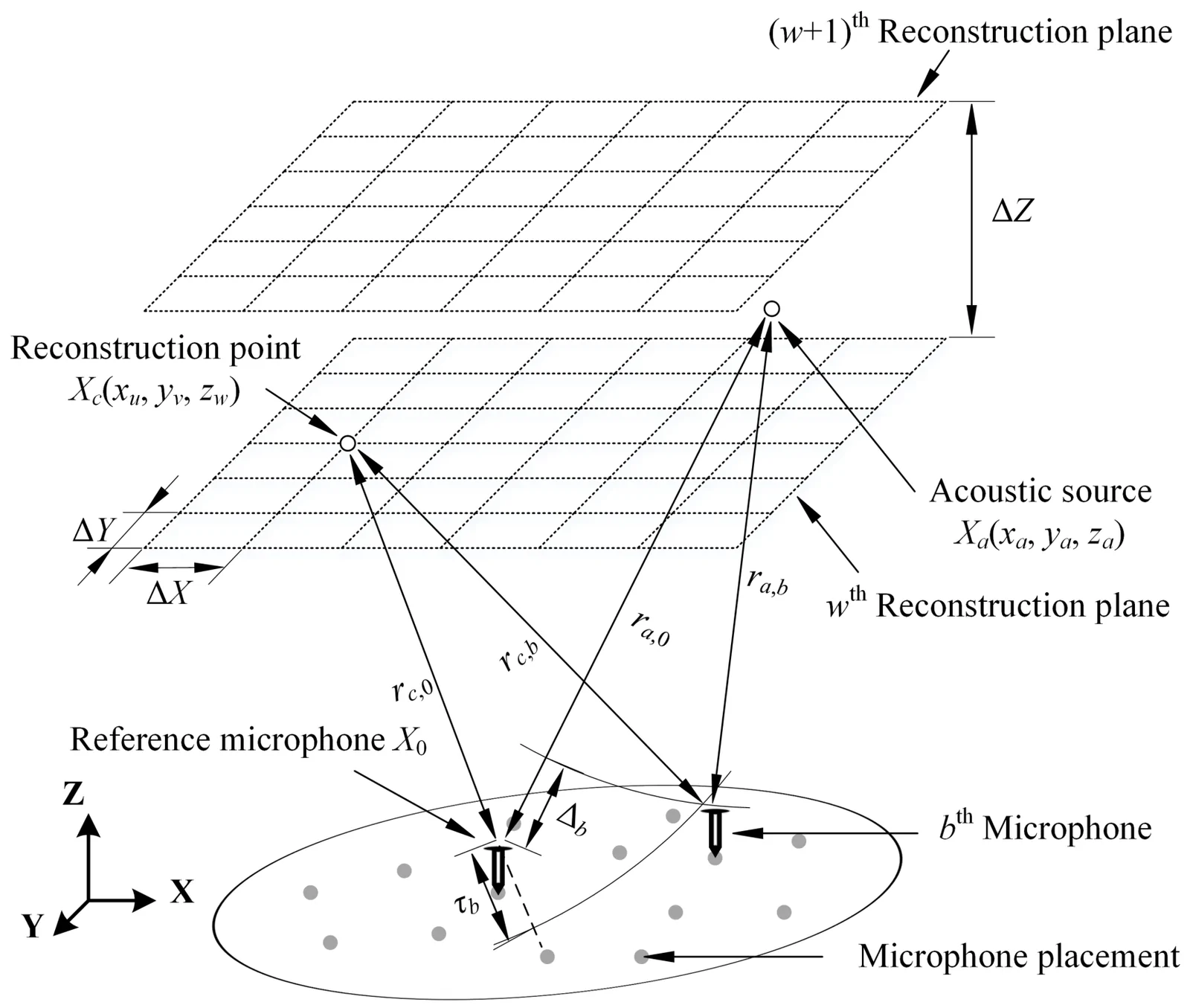
Sketch of setup with a planar microphone array and the slice reconstruction space of interest.

**Figure 2:**
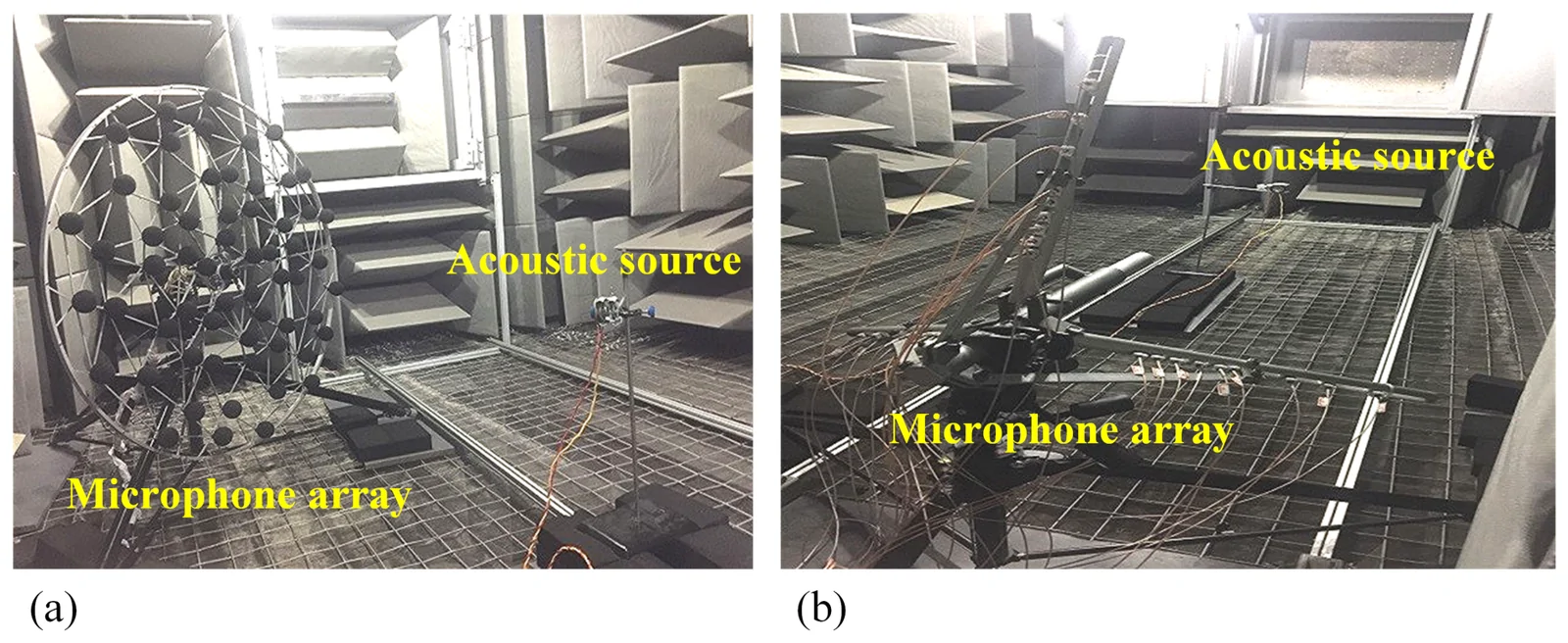
Experimental setup: (**a**) the two-dimensional Brüel & Kjær WA-1558 sliced wheel array (array-I); (**b**) the self-developed depth-direction array (array-II).

**Figure 3:**
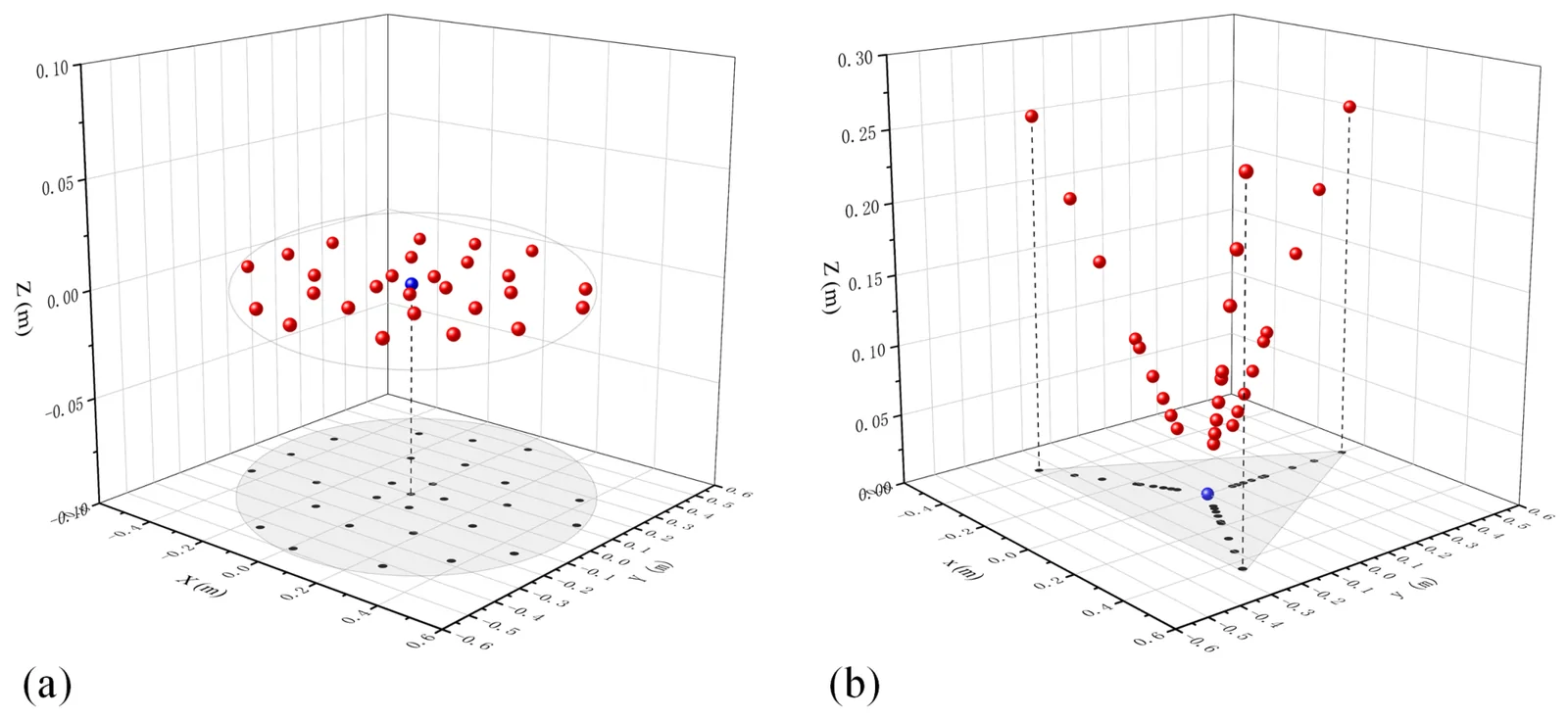
Microphone arrangements in (**a**) array-I and (**b**) array-II. The shaded region indicates the projection of the array onto the *X*-*Y* plane, the red dots represent the microphone sensors, and the blue dot denotes the reference one.

**Figure 4:**
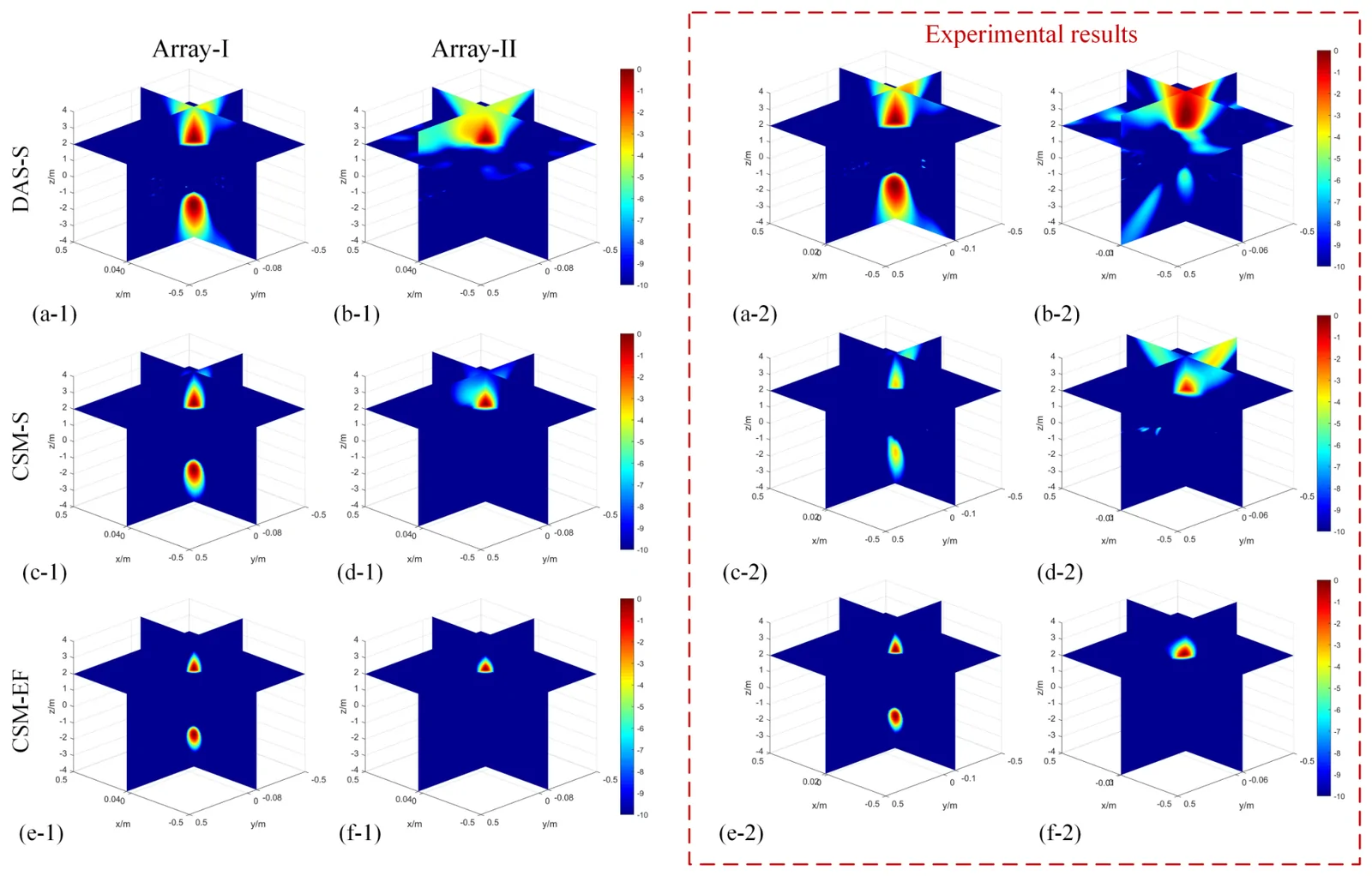
Three-dimensional acoustic imaging of the power-level contribution of a single point source at the assumed position A=(0.04,−0.08,2)m at f=4500Hz: (**a-1**,**b-1**) DAS-S; (**c-1** **d-1**) CSM-S; (**e-1**,**f-1**) CSM-EF, where the first column (**a-1**,**c-1**,**e-1**) represents the results of Array-I, and the second column (**b-1**,**d-1**,**f-1**) denotes the results of Array-II. The panels on the left show the numerical simulations, and the corresponding experimental results are shown in the red boxes on the right (**a-2**–**f-2**).

**Figure 5:**
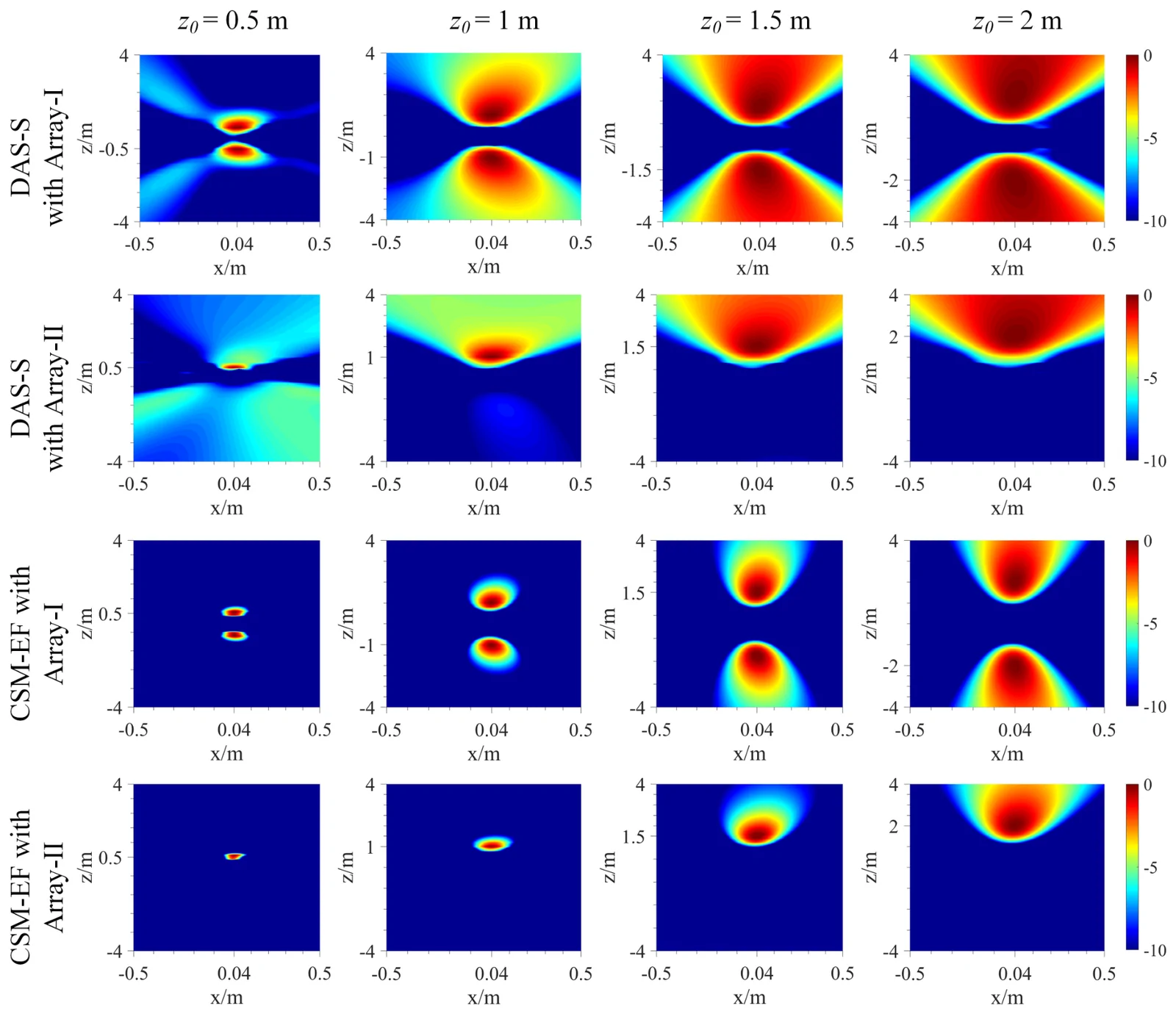
Comparison of the DAS-S and CSM-EF results in the *X*-*Z* plane obtained with Array-I and Array-II. The frequency is f=1500Hz, and the source depths are $z_{0}=0.5\;m$, 1.0m, 1.5m, and 2.0m.

**Figure 6:**
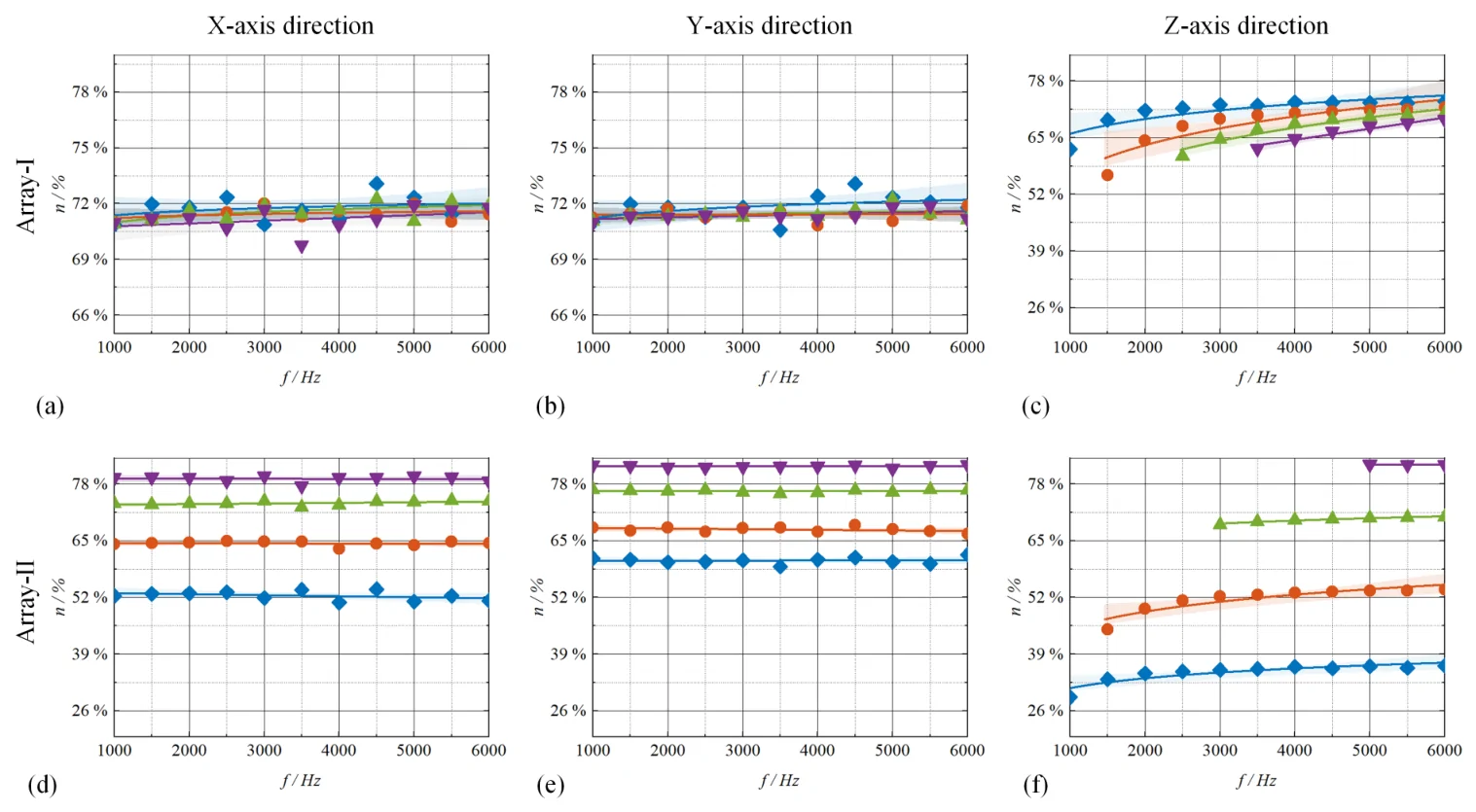
Relative HPBW of CSM-S obtained with array-I and array-II: array-I in the (**a**) *X*-, (**b**) *Y*-, and (**c**) *Z*-directions; array-II in the (**d**) *X*-, (**e**) *Y*-, and (**f**) *Z*-directions. The sound source depths of $z_{0}=0.5\;m$, 1.0m, 1.5m, and 2.0m are marked in blue 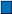, orange 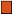, green 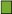, and purple 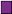, respectively. The scatter points denote the experimental results, while the curves represent the simulation results. The shaded areas represent the confidence intervals of the experimental data.

**Figure 7:**
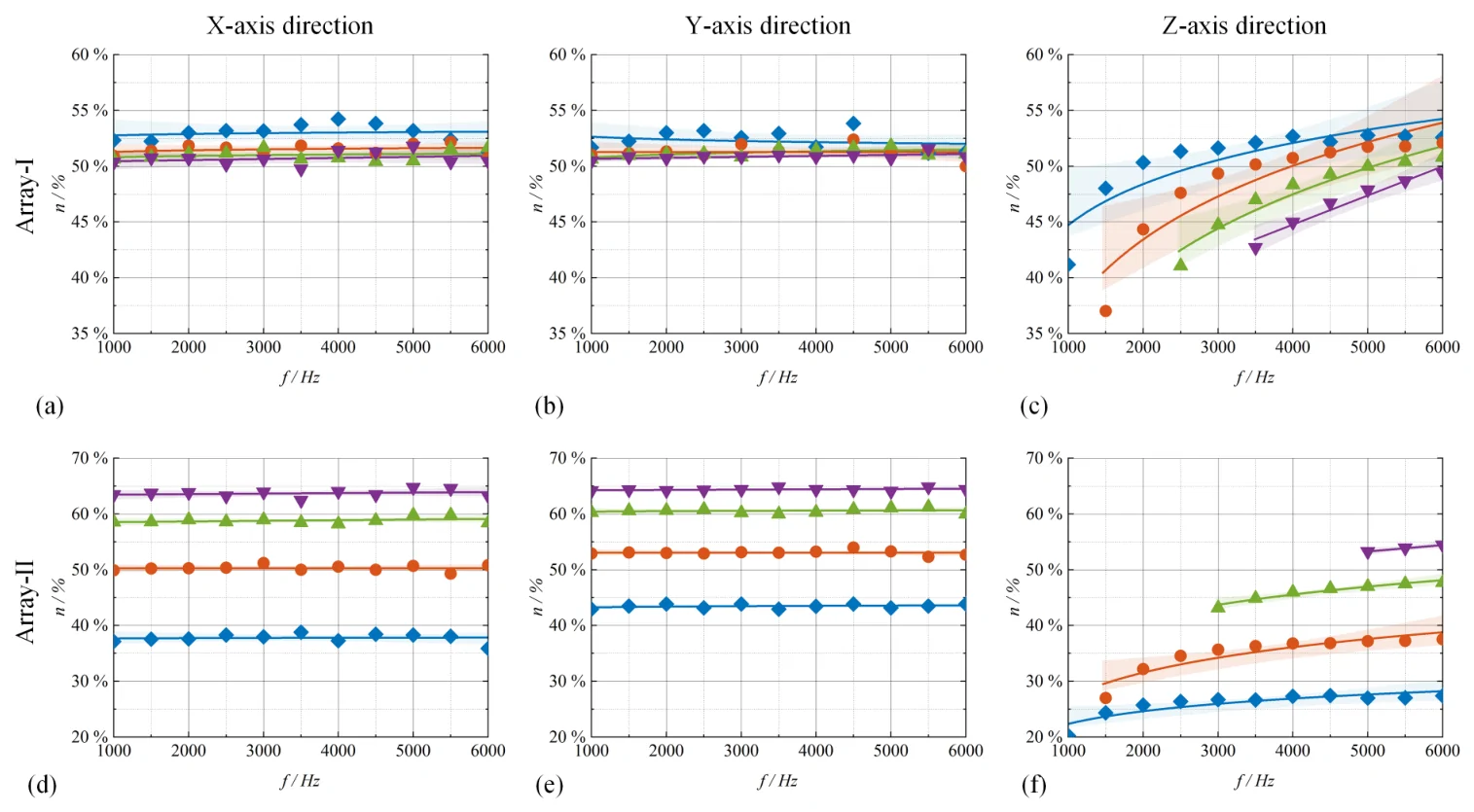
Relative HPBW of CSM-EF obtained with array-I and array-II: array-I in the (**a**) *X*-, (**b**) *Y*-, and (**c**) *Z*-directions; array-II in the (**d**) *X*-, (**e**) *Y*-, and (**f**) *Z*-directions. The sound source depths of $z_{0}=0.5\;m$, 1.0m, 1.5m, and 2.0m are marked in blue 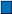, orange 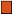, green 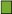, and purple 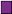, respectively. The scatter points denote the experimental results, while the curves represent the simulation results. The shaded areas represent the confidence intervals of the experimental data.

**Figure 8:**
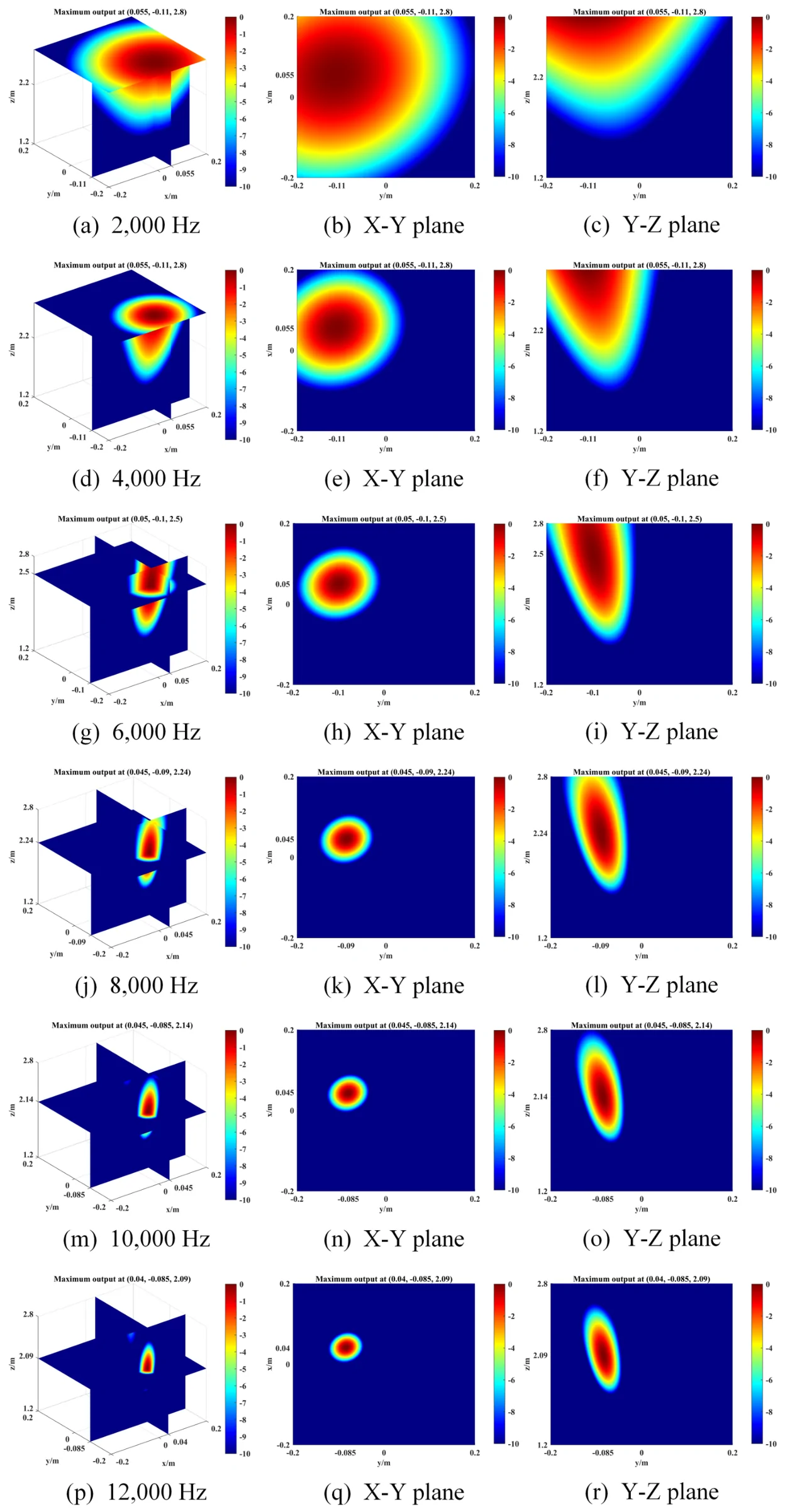
Localization imaging results of the CSM-u_II_ algorithm at frequencies from 2kHz to 12kHz.

**Figure 9:**
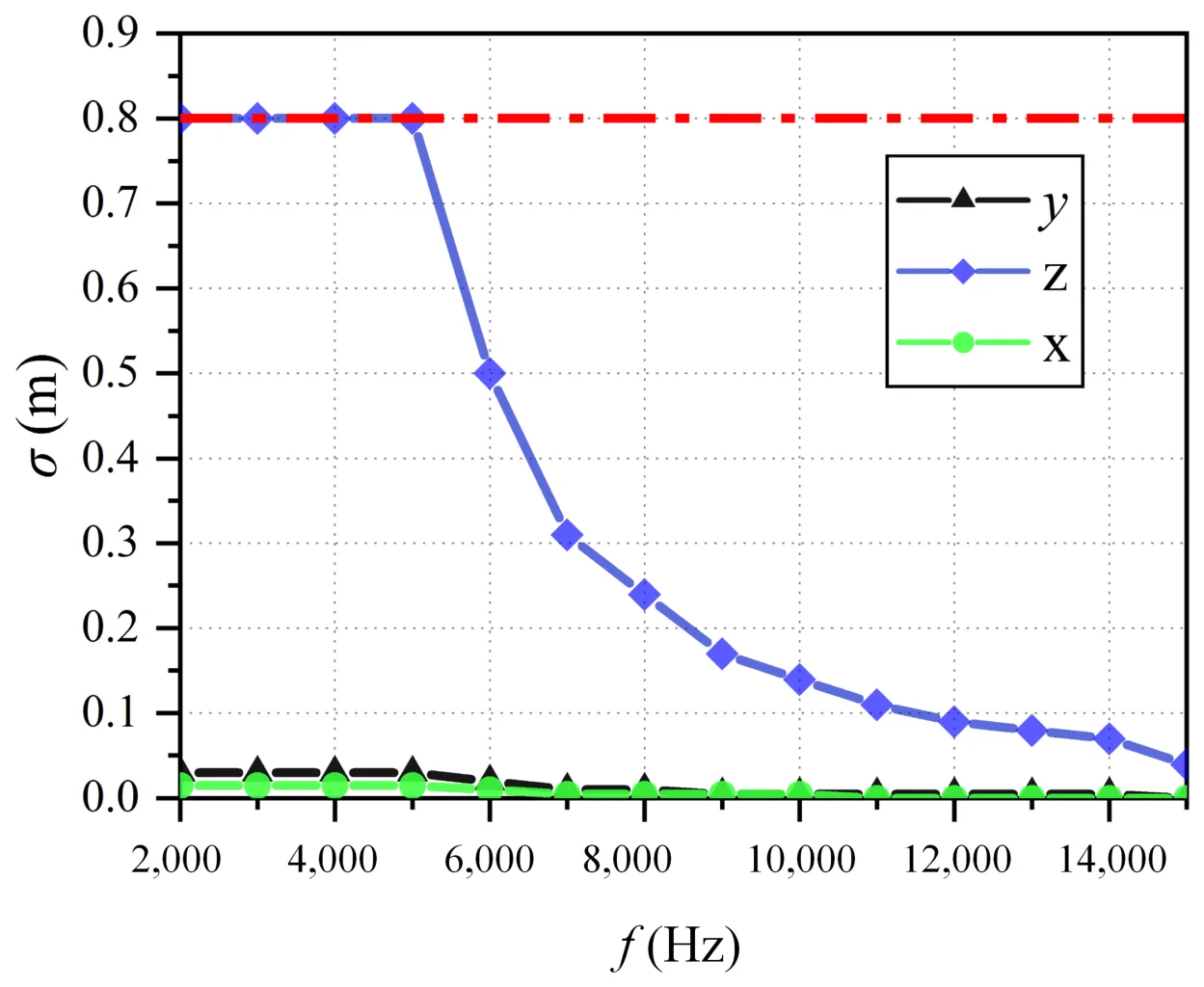
Relative localization errors of the CSM-u_II_ algorithm in the *X*-, *Y*-, and *Z*-directions.

**Figure 10:**
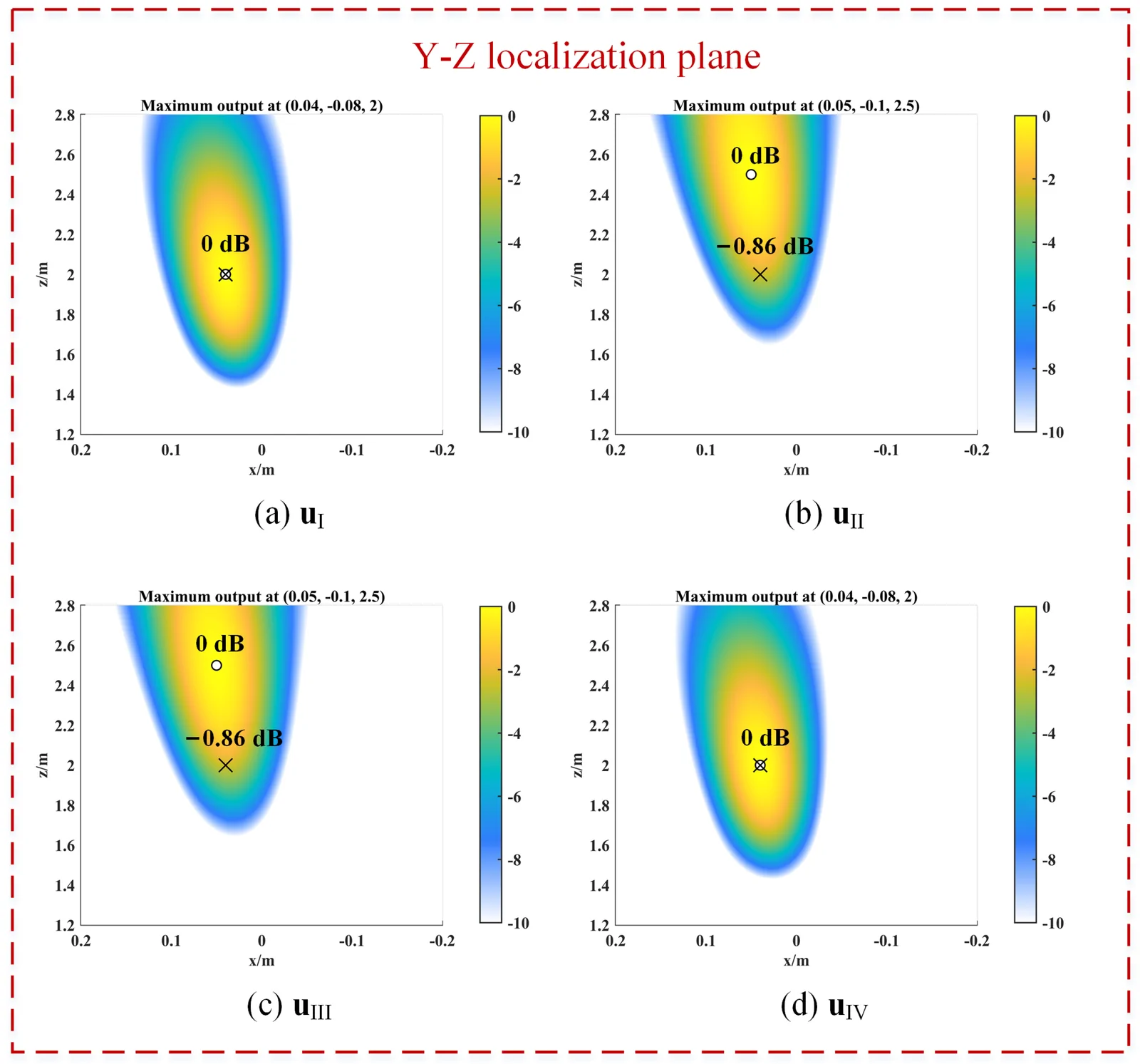
Single-source imaging results of the CSM-S algorithm combined with the four steering vectors on the *Y*-*Z* localization plane.

**Figure 11:**
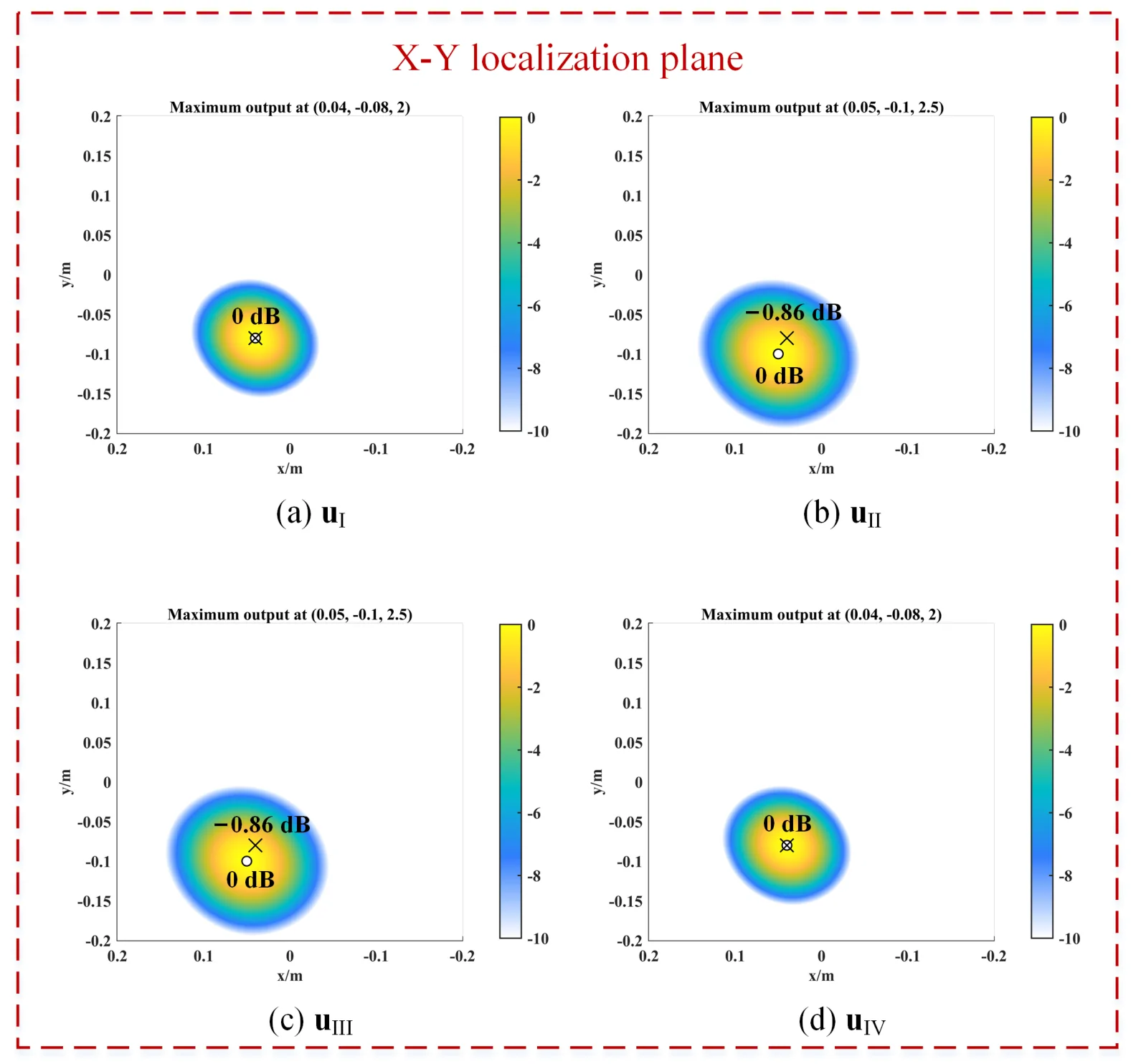
Single-source imaging analysis of CSM-S algorithm combined with four steering vectors; X-Y localization plane.

**Figure 12:**
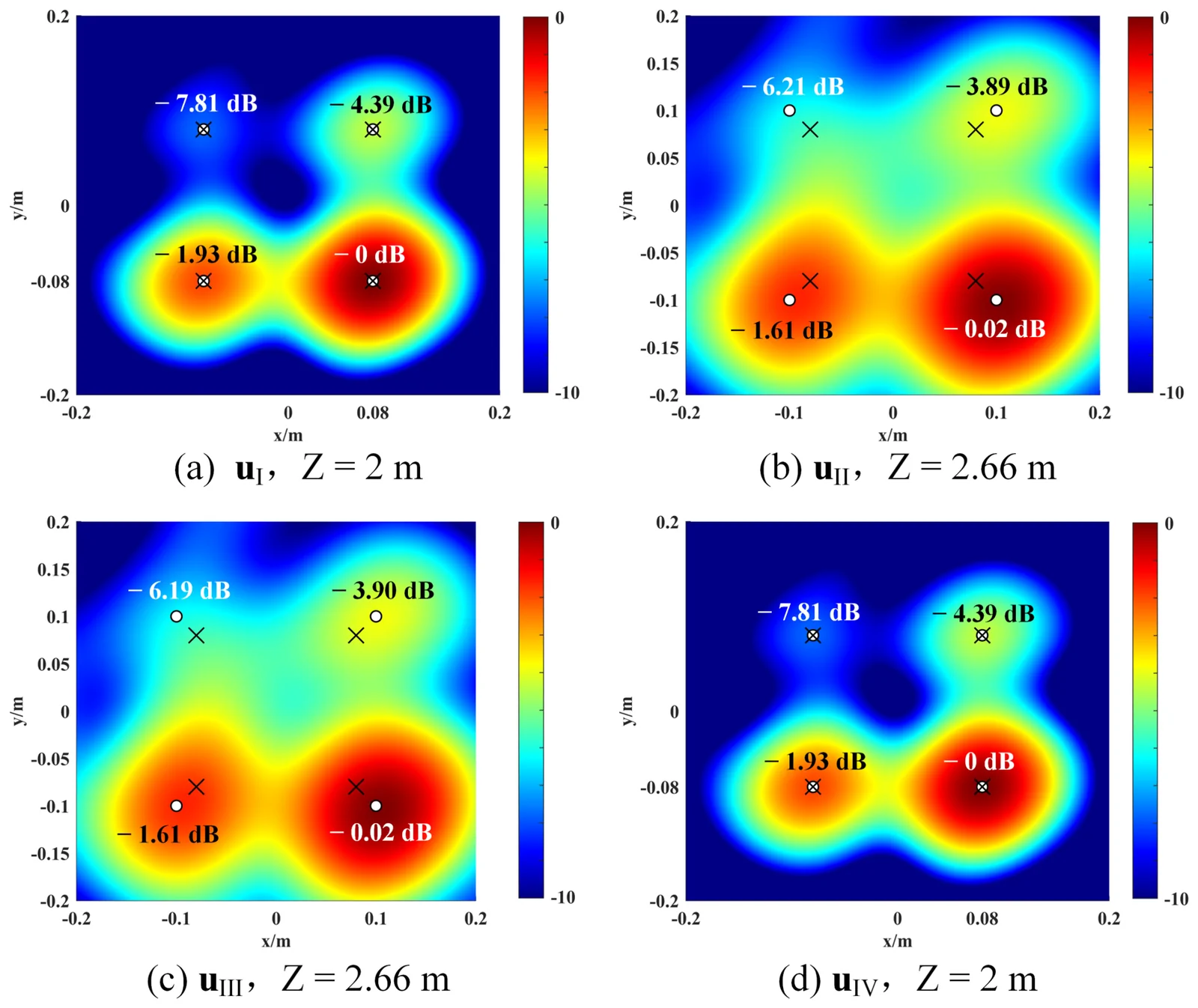
Multi-source imaging results of the CSM-S algorithm combined with the four steering vectors.

**Table 1:** Coordinates of the 27 microphones in array-I (m). Array-I is planar, so z=0 for all microphones.

No.	*x*	*y*	No.	*x*	*y*	No.	*x*	*y*
1	0.0917	0.0317	10	−0.2918	0.3840	19	0.0202	−0.2759
2	0.2501	0.1083	11	0.0363	0.4878	20	−0.1516	−0.4366
3	0.1459	0.2351	12	−0.0928	0.0283	21	0.0104	−0.4822
4	0.3792	0.2641	13	−0.2562	−0.1045	22	0.0585	−0.0774
5	0.4751	0.1162	14	−0.4621	0.0092	23	0.1803	−0.2043
6	−0.0018	0.0970	15	−0.4554	−0.1589	24	0.2687	−0.0661
7	−0.0257	0.2713	16	−0.4527	0.1852	25	0.3684	−0.2791
8	−0.1785	0.2114	17	−0.0555	−0.0795	26	0.4618	−0.1392
9	−0.1340	0.4423	18	−0.1386	−0.2346	27	0.2574	−0.4159

**Table 2:** Array-II coordinates of 27 microphones (m).

No.	*x*	*y*	*z*	No.	*x*	*y*	*z*	No.	*x*	*y*	*z*
1	0.0774	−0.0447	0.0448	10	0	0.0894	0.0448	19	−0.0774	−0.0447	0.0448
2	0.0929	−0.0537	0.0537	11	0	0.1073	0.0537	20	−0.0929	−0.0537	0.0537
3	0.1130	−0.0652	0.0653	12	0	0.1305	0.0653	21	−0.1130	−0.0652	0.0653
4	0.1389	−0.0802	0.0804	13	0	0.1604	0.0804	22	−0.1389	−0.0802	0.0804
5	0.1726	−0.0996	0.0998	14	0	0.1993	0.0998	23	−0.1726	−0.0996	0.0998
6	0.1827	−0.1055	0.1057	15	0	0.2110	0.1057	24	−0.1827	−0.1055	0.1057
7	0.2725	−0.1574	0.1576	16	0	0.3147	0.1576	25	−0.2725	−0.1574	0.1576
8	0.3456	−0.1995	0.1999	17	0	0.3990	0.1999	26	−0.3456	−0.1995	0.1999
9	0.4402	−0.2541	0.2546	18	0	0.5083	0.2546	27	−0.4402	−0.2541	0.2546

**Table 3:** Data-acquisition and CSM-processing parameters of the experiments.

Parameter	Value
Acquisition system	Brüel & Kjær PULSE (PortablePULSE format)
Front-end/channels	60-channel front-end, common master clock; 27 channels (Table 1 and Table 2) used for processing
Sampling frequency $f_{s}$	32,768Hz
Effective analysis bandwidth	12.8kHz (2.56× oversampling)
ADC word length	16-bit integer
Tone gating	Rectangular on/off envelope, repetition period 10s
Record length per test case	165,888 samples per channel (5.0625s), within the steady-state portion of the tone
FFT block size $N_{\mathrm{FFT}}$	512 (Δf=64Hz)
Window	Hanning
CSM estimator	Single snapshot (K=1), Equation (12), block taken from the central portion of the record; diagonal retained
Microphone calibration	B&K Type 4231 calibrator, 94dB @ 1kHz, per channel before each session
Repeated measurements	3 complete repetitions of the experiment (both arrays, all source positions, all 11 frequencies)

**Table 4:** Computation times of DAS-S, CSM-S, and CSM-EF with array-I and array-II under identical reconstruction conditions for two grid densities.

Grid Points	DAS-S with Array-I	DAS-S with Array-II	CSM-S with Array-I	CSM-S with Array-II	CSM-EF with Array-I	CSM-EF with Array-II
85,833	0.42 s	0.26 s	104.03 s	104.75 s	93.88 s	92.75 s
418,241	0.76 s	0.81 s	457.57 s	471.55 s	457.63 s	460.57 s

**Table 5:** Sound power measurement results of proposed sound source point $\left(x_{a}=x_{c}\right)$ positions of different steering vector algorithms.

Algorithm Model	$I_{\max}^{2}$ (a.u.)	$I_{c}^{2}$ (a.u.)	$B_{c}$ (dB)
CSM-u_I_	603.712	603.712	0
CSM-u_II_	3047.0	2500	−0.86
CSM-u_III_	3047.7	2500	−0.86
CSM-u_IV_	603.805	603.805	0

**Figure A1:**
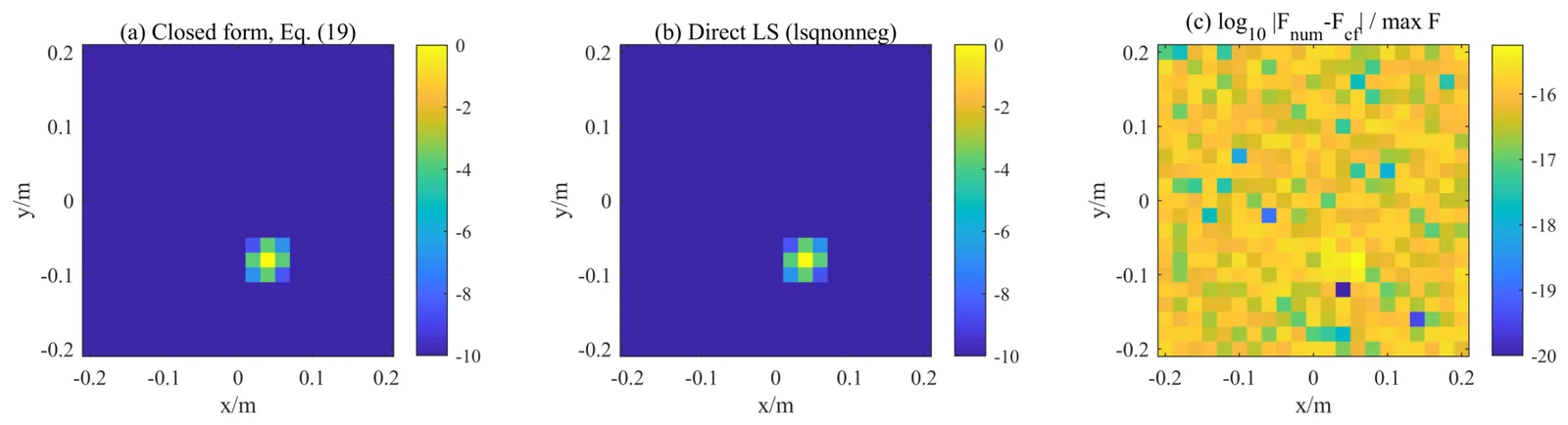
CSM-EF output on the z=2.00 m slice through the source, normalized to the maximum (dB): (**a**) closed-form solution, Equation (20); (**b**) direct numerical minimization of Equation (17) by NNLS; (**c**) $\log_{10}$ of the pointwise difference $|F_{\mathrm{num}}-F|/\max F$, which lies at the $10^{-16}$ level (machine precision).

## Data Availability

The original contributions presented in this study are included in the article. Further inquiries can be directed to the corresponding author.

## Appendix A. Numerical Verification of the Closed-Form CSM-EF Solution

### Appendix A.1. Verification Procedure

To verify numerically that Equation (20) is the exact solution of the least-squares matching problem defined in Equation (17), the minimization

(A1) $$\min_{a\geq 0}\;{∥R-aH∥}_{F}^{2}$$

was solved by direct numerical optimization, without using the closed-form expressions in Equations (19) and (20), and the results were compared with the closed-form solution point by point over the entire scan grid. The simulated configuration in Section 3 was used: a single monopole source of strength *A* ($A^{2}=2500$) located at (0.04,−0.08,2.00) m, B=27 microphones, f=10 kHz, and a scan grid of 21×21×33=14,553 points (Δx=Δy=0.02 m, Δz=0.05 m, z∈[1.2,2.8] m). The measured CSM was formed as $R=pp^{H}$ from the simulated microphone pressures. Since

(A2) R−aHF2=r−ah22,r=RevecRImvecR∈R2B2,h=RevecHImvecH,

problem (A1) is an ordinary real-valued nonnegative least-squares problem in the single unknown *a*. At every grid point, it was solved with the active-set NNLS algorithm (MATLAB R2023b, lsqnonneg), yielding $a_{\mathrm{num}}^{*}$. The corresponding minimum error $E_{\mathrm{num}}^{\min}={∥R-a_{\mathrm{num}}^{*}H∥}_{F}^{2}$ was obtained by substituting $a_{\mathrm{num}}^{*}$ back into Equation (17), and the resulting output $F_{\mathrm{num}}={∥R∥}_{F}^{2}-E_{\mathrm{num}}^{\min}$ was compared with Equation (20). As an independent second check, Equation (A1) was also minimized by a derivative-free bounded one-dimensional search (MATLAB fminbnd, $\mathrm{TolX}=10^{-12}$).

### Appendix A.2. Results

The results are summarized in Table A1 and Figure A1. The numerically obtained $a_{\mathrm{num}}^{*}$, $E_{\mathrm{num}}^{\min}$ and $F_{\mathrm{num}}$ agree with the closed-form expressions to within $10^{-15}$ relative deviation over the entire grid, i.e., at double-precision machine accuracy, and both approaches locate the maximum at the prescribed source position. The numerical solution is strictly positive at all grid points, which confirms that the constraint a≥0 in Equation (A1) is never active, as established analytically in Section 2.3 from ${\langle H,R\rangle }_{F}=g^{H}Rg\geq 0$. At the true source position, the recovered scale parameter equals the prescribed $A^{2}$ to within double precision. These results confirm that Equation (20) is the exact solution of the least-squares problem in Equation (17); all CSM-EF results in Section 3 and Section 4 were computed from Equation (20) and are therefore unaffected.

**Table A1:** Comparison between the closed-form CSM-EF solution, Equations (19) and (20), and the direct numerical minimization of Equation (17) over all 14,553 grid points (noise-free simulation, B=27, f=10 kHz, source at (0.04,−0.08,2.00) m).

Quantity	NNLS (lsqnonneg)	Bounded Search (fminbnd) ^1^
$\max_{\mathrm{grid}}\;\left|a_{\mathrm{num}}^{*}-a^{*}\right|/\max a^{*}$	$1.0\times 10^{-15}$	$2.7\times 10^{-8}$
$\max_{\mathrm{grid}}\;|E_{\mathrm{num}}^{\min}-E_{c}^{\min}{|/∥R∥}_{F}^{2}$	$8.5\times 10^{-16}$	-
$\max_{\mathrm{grid}}\;\left|F_{\mathrm{num}}-F\right|/\max F$	$9.0\times 10^{-16}$	-
$\min_{\mathrm{grid}}\;a_{\mathrm{num}}^{*}$	$2.6\times 10^{-3}$ (>0)	-
$a^{*}$ at the true source position (prescribed $A^{2}=2500$)	2500 (rel. error $<10^{-15}$)	2500 (rel. error $<10^{-8}$)
Location of maximum output (m)	(0.04,−0.08,2.00)	(0.04,−0.08,2.00)

^1^ Only $a^{*}$ and the derived quantities were compared. The $10^{-8}$ level is the $\sqrt{\varepsilon }$ resolution limit of function-value-based minimization of a quadratic function and is not an error of Equation (19).

## Appendix B. Absolute Half-Power Beam Widths

Table A2, Table A3 and Table A4 list the absolute HPBW of the DAS-S reference for array-I and array-II in the *X*-, *Y*- and *Z*-directions at the four source depths and eleven frequencies used in Section 4.3 and Section 4.4; the array-I values constitute the denominator ${\mathrm{HPBW}}_{1}$ of the relative width *n*. Table A5 and Table A6 list the absolute *Z*-direction HPBW of CSM-S and CSM-EF for both arrays, so that all *Z*-direction values entering Figure 6c,f and Figure 7c,f are available in absolute form; the *X*- and *Y*-direction values of CSM-S and CSM-EF follow as $n\cdot {\mathrm{HPBW}}_{1}$ from Table A2 and Table A3. In all tables, “-” denotes a right-censored case in which the −3 dB level is not reached within the *Z*-scanning region, i.e., only HPBW>2.6 m can be stated.

**Table A2:** Absolute HPBW (m) of DAS-S in the *X*-direction.

	Array-I (=${\mathrm{HPBW}}_{1}$)				Array-II			
f (Hz)	0.5 m	1.0 m	1.5 m	2.0 m	0.5 m	1.0 m	1.5 m	2.0 m
1000	0.237	0.383	0.543	0.709	0.1785	0.3995	0.6630	0.9362
1500	0.157	0.253	0.359	0.469	0.1175	0.2616	0.4334	0.6115
2000	0.118	0.189	0.269	0.351	0.0877	0.1950	0.3229	0.4555
2500	0.094	0.152	0.214	0.280	0.0700	0.1556	0.2575	0.3633
3000	0.078	0.126	0.178	0.234	0.0583	0.1294	0.2143	0.3023
3500	0.067	0.108	0.154	0.200	0.0499	0.1108	0.1835	0.2588
4000	0.058	0.094	0.134	0.174	0.0436	0.0969	0.1605	0.2264
4500	0.052	0.084	0.119	0.156	0.0388	0.0861	0.1426	0.2011
5000	0.047	0.076	0.107	0.140	0.0349	0.0775	0.1283	0.1810
5500	0.042	0.068	0.098	0.127	0.0317	0.0704	0.1166	0.1645
6000	0.039	0.063	0.090	0.116	0.0292	0.0645	0.1069	0.1508

**Table A3:** Absolute HPBW (m) of DAS-S in the *Y*-direction.

	Array-I (=${\mathrm{HPBW}}_{1}$)				Array-II			
f (Hz)	0.5 m	1.0 m	1.5 m	2.0 m	0.5 m	1.0 m	1.5 m	2.0 m
1000	0.237	0.393	0.549	0.717	0.2433	0.4370	0.6908	0.9637
1500	0.157	0.255	0.363	0.474	0.1571	0.2846	0.4505	0.6263
2000	0.118	0.191	0.272	0.354	0.1166	0.2118	0.3353	0.4663
2500	0.094	0.152	0.217	0.284	0.0928	0.1688	0.2674	0.3718
3000	0.079	0.127	0.181	0.236	0.0771	0.1404	0.2224	0.3093
3500	0.068	0.109	0.155	0.202	0.0660	0.1202	0.1904	0.2648
4000	0.059	0.095	0.135	0.177	0.0577	0.1051	0.1665	0.2316
4500	0.052	0.085	0.121	0.157	0.0513	0.0934	0.1479	0.2058
5000	0.047	0.077	0.109	0.141	0.0461	0.0840	0.1331	0.1852
5500	0.043	0.069	0.099	0.129	0.0419	0.0763	0.1210	0.1683
6000	0.039	0.063	0.091	0.117	0.0384	0.0700	0.1109	0.1543

**Table A4:** Absolute HPBW (m) of DAS-S in the *Z*-direction. “-”: the −3 dB crossing lies outside the *Z*-scanning region (right-censored, HPBW>2.6 m); *n* is undefined for these cases.

	Array-I (=${\mathrm{HPBW}}_{1}$)				Array-II			
f (Hz)	0.5 m	1.0 m	1.5 m	2.0 m	0.5 m	1.0 m	1.5 m	2.0 m
1000	1.094	–	–	–	0.5004	–	–	–
1500	0.608	2.204	–	–	0.2947	2.2910	–	–
2000	0.432	1.316	–	–	0.2129	1.1577	–	–
2500	0.337	0.964	2.342	–	0.1675	0.8076	–	–
3000	0.277	0.768	1.744	–	0.1383	0.6298	2.3945	–
3500	0.236	0.642	1.405	2.511	0.1180	0.5199	1.7695	–
4000	0.206	0.552	1.184	2.219	0.1029	0.4443	1.4218	–
4500	0.182	0.486	1.027	1.880	0.0912	0.3887	1.1970	–
5000	0.164	0.433	0.907	1.637	0.0820	0.3460	1.0381	2.5238
5500	0.148	0.392	0.815	1.454	0.0744	0.3120	0.9191	2.1499
6000	0.136	0.358	0.739	1.309	0.0682	0.2843	0.8261	1.8808

**Table A5:** Absolute *Z*-direction HPBW (m) of CSM-S. “-”: −3 dB level not reached within the *Z*-scanning region (HPBW>2.6 m). ^†^: the DAS-S array-I reference is censored at this point (Table A4), so *n* is undefined and the point is absent from Figure 6; here, n<HPBW/2.6 m. ^‡^: the reference is resolved (2.219 m) but CSM-S is censored, i.e., n>117%; see text.

	Array-I				Array-II			
f (Hz)	0.5 m	1.0 m	1.5 m	2.0 m	0.5 m	1.0 m	1.5 m	2.0 m
1000	0.6562	2.2453 ^†^	–	–	0.1647	–	–	–
1500	0.4126	1.2281	–	–	0.1082	1.0391	–	–
2000	0.3035	0.8448	1.8535 ^†^	–	0.0800	0.5490	–	–
2500	0.2368	0.6360	1.3915	2.5507 ^†^	0.0612	0.3978	1.8530	–
3000	0.1952	0.5191	1.0864	2.0650 ^†^	0.0523	0.3283	1.4407	–
3500	0.1683	0.4459	0.9441	1.6715 ^†^	0.0465	0.2728	1.1664	–
4000	0.1481	0.3883	0.8075	1.4416	0.0389	0.2355	0.9761	– ^‡^
4500	0.1304	0.3407	0.6996	1.2451	0.0322	0.2119	0.8442	2.4058
5000	0.1176	0.3076	0.6300	1.1120	0.0319	0.1869	0.7306	2.0267
5500	0.1056	0.2779	0.5656	1.0046	0.0273	0.1709	0.6684	1.7629
6000	0.0975	0.2528	0.5156	0.9021	0.0249	0.1589	0.5957	1.5439

**Table A6:** Absolute *Z*-direction HPBW (m) of CSM-EF. Notation as in Table A5; ^†^ marks points absent from Figure 7 because the DAS-S array-I reference is censored.

	Array-I				Array-II			
f (Hz)	0.5 m	1.0 m	1.5 m	2.0 m	0.5 m	1.0 m	1.5 m	2.0 m
1000	0.4506	1.2714 ^†^	–	–	0.1015	1.0680 ^†^	–	–
1500	0.2920	0.8160	1.7274 ^†^	–	0.0717	0.6185	–	–
2000	0.2175	0.5836	1.2257 ^†^	2.2275 ^†^	0.0548	0.3727	–	–
2500	0.1730	0.4590	0.9620	1.6814 ^†^	0.0442	0.2790	1.4509	–
3000	0.1430	0.3790	0.7805	1.3796 ^†^	0.0369	0.2247	1.0333	–
3500	0.1230	0.3220	0.6605	1.0669 ^†^	0.0315	0.1887	0.7941	–
4000	0.1085	0.2800	0.5725	0.9980	0.0281	0.1634	0.6539	2.0489
4500	0.0950	0.2490	0.5060	0.8780	0.0251	0.1432	0.5583	1.6154
5000	0.0865	0.2240	0.4535	0.7840	0.0221	0.1286	0.4882	1.3444
5500	0.0780	0.2030	0.4110	0.7080	0.0201	0.1162	0.4364	1.1592
6000	0.0715	0.1865	0.3755	0.6465	0.0187	0.1067	0.3941	1.0237
